# A foundation model for sleep-based risk stratification and clinical outcomes

**DOI:** 10.1038/s41467-026-75326-9

**Published:** 2026-08-03

**Authors:** Erhan Bilal, Matheus Lima Diniz Araujo, Kristen L. Beck, Catherine M. Heinzinger, Samer Ghosn, Carl Y. Saab, Nancy Foldvary-Schaefer, Jeffrey L. Rogers, Reena Mehra

**Affiliations:** 1 Digital Health, IBM Research, T.J. Watson Research Center, Yorktown Heights, NY USA; 2 Sleep Disorders Center, Neurological Institute Cleveland Clinic Foundation, Cleveland, OH USA; 3 Digital Health, IBM Research, Almaden Research Center, San Jose, CA USA; 4 Department of Biomedical Engineering, Cleveland Clinic Foundation, Cleveland, OH USA; 5 School of Medicine, Case Western Reserve University, Cleveland, OH USA; 6 Department of Engineering, Brown University, Providence, RI USA; 7 Sleep Disorders & Epilepsy Centers, Neurological Institute Cleveland Clinic Foundation, Cleveland, OH USA; 8 Department of Neurosurgery, Yale School of Medicine, Yale University, New Haven, CT USA; 9 Division of Pulmonary, Critical Care, and Sleep Medicine, Department of Medicine, University of Washington, Seattle, WA USA

**Keywords:** Risk factors, Translational research, Machine learning, Sleep, Sleep disorders

## Abstract

Clinical sleep studies capture multiple physiologic signals, yet interpretation is often reduced to single summary measures of limited prognostic value, such as the apnea–hypopnea index. We present a foundation model that learns rich representations of sleep physiology from more than 10,000 clinical sleep recordings linked to electronic medical records. Here we show that sleep physiology contains latent risk structure invisible to conventional metrics, identifying five patient risk groups with markedly different trajectories for mortality, cardiovascular, and neurological disease. The highest-risk group shows more than double the mortality risk of the lowest, whereas apnea–hypopnea index severity categories show limited predictive value. The framework generalizes to the independent Sleep Heart Health Study, distinguishing high- and low-risk patients despite lower-resolution data. We demonstrate that foundation models recover clinically meaningful risk information embedded in routine sleep recordings that conventional metrics systematically miss, providing a scalable path to precision sleep medicine.

## Abstract (web summary)

Clinical sleep studies generate rich physiologic data, yet traditional metrics capture only part of their predictive value. Here, the authors show that a foundation model trained on thousands of recordings learns latent sleep patterns that cluster patients into clinically meaningful risk groups.

## Introduction

Sleep is fundamental to health and well-being. Its disruption by common disorders diminishes quality of life and increases the risk of cardiovascular, neurologic, and psychiatric disease^1^. Sleep apnea alone affects nearly one billion people worldwide, underscoring the scope of this public health burden^2^. Yet, assessment of sleep integrity remains limited because clinical interpretation of polysomnography (PSG), the gold-standard diagnostic test, is often reduced to the apnea-hypopnea index (AHI)^3^. While AHI is central to current diagnostic criteria for sleep-disordered breathing as defined by the American Academy of Sleep Medicine (AASM ICSD-3-TR), it captures only a narrow slice of sleep physiology and carries limited prognostic value. Identifying effective ways to incorporate the full richness of PSG time-series data, together with electronic medical records (EMRs), holds the potential to move beyond these constraints and deliver scalable innovations in precision sleep medicine^4^.

Attempts to overcome these limitations have included cluster analysis of PSG-derived indices, such as AHI, arousal index, and sleep stage proportions^5–7^. While informative, these approaches rely on technologist-scored aggregate measures that obscure the dynamic complexity of sleep physiology. Other efforts have focused on specific physiologic burdens, such as hypoxic burden or heart rate arousal response, which may capture particular aspects of sleep-disordered breathing but remain narrowly focused and fail to capture the broader physiologic landscape of sleep^8^.

Artificial intelligence (AI) has shown promise in revealing hidden disease phenotypes in other areas of medicine, such as metabolic subtypes in diabetes^9^ and clinical subgroups in Alzheimer’s disease^10^. These traditional machine learning models often depend on hand-crafted features and task-specific designs, limiting their ability to generalize across populations or adapt to new datasets. Foundation models, a class of AI architectures trained on large, diverse datasets, offer a scalable alternative. By learning broad, general-purpose physiologic representations, they can be adapted with minimal additional training to downstream tasks^11^. Extending this methodology to PSG data, however, presents unique challenges, since sleep recordings consist of multimodal time-series signals that vary in length and sampling frequency. Developing effective methods to represent and tokenize such data remains an open area of research.

Here we introduce a foundation model for sleep health, designed to uncover latent physiologic patterns from full-night PSG studies and link them with long-term clinical outcomes. Leveraging a unique resource of 10,000 high-resolution PSG studies from the Cleveland Clinic Sleep Signals, Testing, and Reports Linked to Patient Traits (STARLIT) Registry, paired with EMRs spanning more than a decade, we trained a transformer-based model that generates high-dimensional embeddings of sleep physiology^12^. Using clustering, we identified distinct risk groups that stratify patients by cardiovascular and neurologic outcomes and by mortality, outperforming conventional AHI-based severity categories. Finally, external validation in an independent, large, population-based cohort confirmed the generalizability of this framework. Together, these findings establish the potential of foundation models to capture complex physiologic signals, reveal previously unrecognized risk markers, and provide a scalable path toward precision sleep medicine.

## Results

**Fig. 1:**
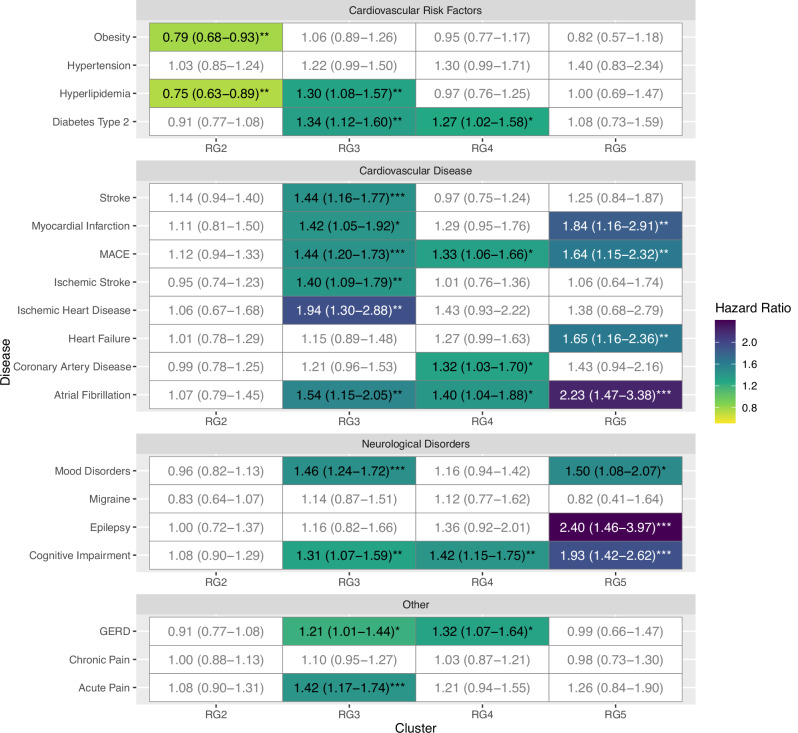
Hazard ratios of disease incidence across embedding-derived risk groups. The highest-risk group (RG5) exhibits elevated incidence of cardiovascular, neurologic, and psychiatric outcomes compared to RG1, with a monotonic increase in risk observed across the five embedding-defined groups (RG1–RG5), demonstrating the prognostic value of embedding-derived clusters beyond conventional sleep metrics. Cox proportional hazards models were adjusted for age, gender, body mass index, and the outcome-specific comorbidities listed in Supplementary Data 3, with risk group 1 (RG1) as the reference group. Values shown are hazard ratios with 95% confidence intervals. Asterisks denote two-sided Wald-test significance thresholds (*p < 0.05, **p < 0.01, ***p < 0.001). Specific Wald *z* statistics and specific two-sided *p* values are provided in Supplementary Data 1. No multiple-comparison adjustment was applied. Abbreviations: MACE, major adverse cardiovascular events; GERD, gastroesophageal reflux disease.

**Fig. 2:**
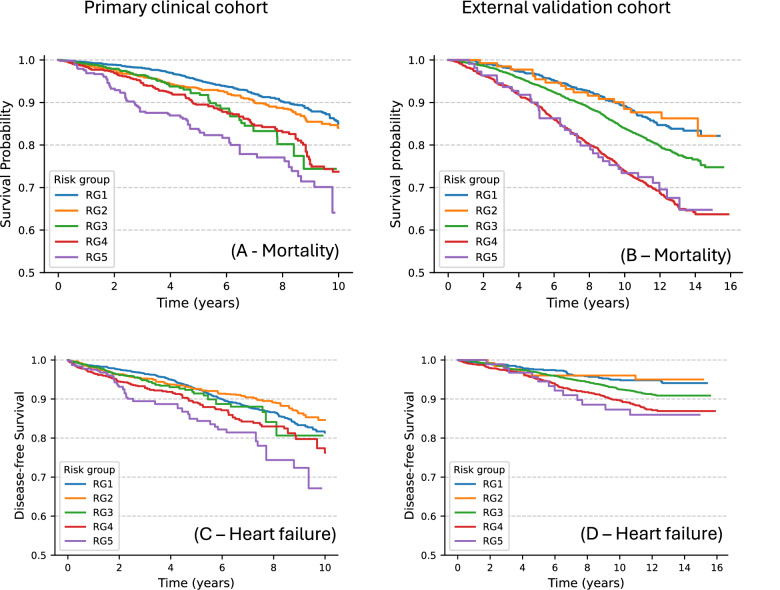
Kaplan–Meier survival curves stratified by embedding-derived risk groups across cohorts. Progressively higher embedding-derived risk groups show graded reductions in survival probability for all-cause mortality and incident heart failure across both the STARLIT-10k clinical cohort and the external SHHS validation cohort, with the strongest separation observed for RG5. Kaplan–Meier curve separation was assessed using two-sided log-rank tests, and hazard ratios were estimated using age- and gender-adjusted Cox proportional hazards models. **A**,**C**: STARLIT-10k clinical cohort (propensity-adjusted); **B**,**D**: SHHS external validation cohort. For mortality, progressively higher risk groups (RG2–RG5) showed significantly increased risk in the STARLIT cohort after adjustment for age and gender (RG2 HR = 1.37, 95% CI = 1.10–1.71, *p* = 0.0058; RG3 HR = 1.51, 95% CI = 1.17–1.96, *p* = 0.0016; RG4 HR = 1.64, 95% CI = 1.26–2.15, *p* = 2.8×10⁻⁴; RG5 HR = 2.71, 95% CI = 1.93–3.81, *p* = 8.5×10⁻⁹). In SHHS, a similar gradient of risk was observed (RG4 HR = 1.31, 95% CI = 1.07–1.62, *p* = 0.0094; RG5 HR = 1.58, 95% CI = 1.07–2.32, *p* = 0.021). For heart failure, the STARLIT cohort showed a significant association for RG5 (HR = 1.67, 95% CI = 1.12–2.48, *p* = 0.011), while SHHS confirmed increased risk for RG5 (HR = 2.13, 95% CI = 1.11–4.09, *p* = 0.023).

**Fig. 3:**
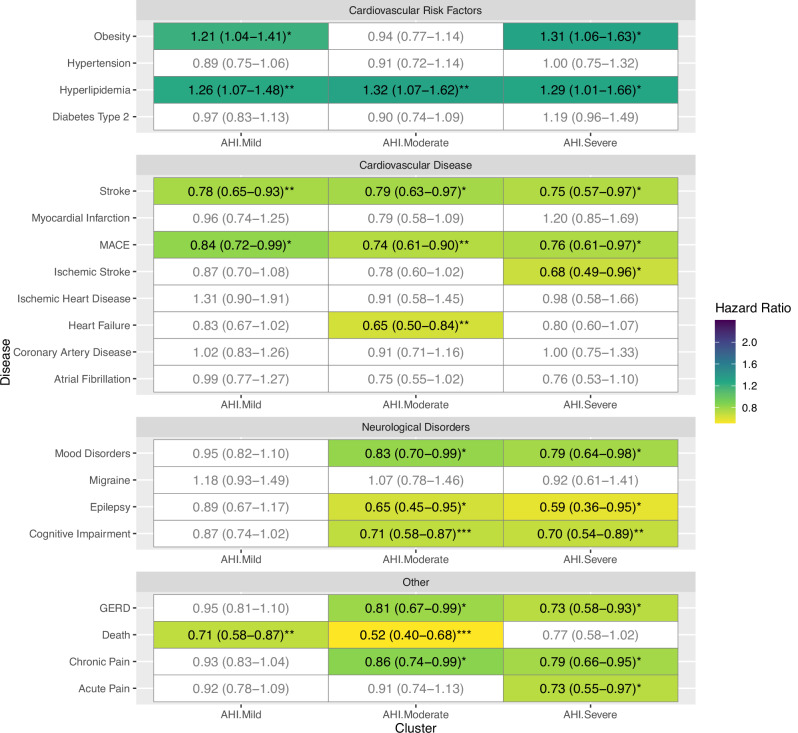
Limited prognostic value of apnea-hypopnea index (AHI) severity categories. Hazard ratios for incident disease and all-cause mortality across AHI-defined strata (mild, moderate, severe) show only limited and inconsistent prognostic separation compared with normal AHI ( < 5), highlighting the limited prognostic utility of conventional AHI-based classification. Cox proportional hazards models used normal AHI ( < 5) as the reference group and were adjusted for age, gender, body mass index, and outcome-specific comorbidities. Values shown are hazard ratios with 95% confidence intervals. Asterisks denote two-sided Wald-test significance thresholds (*p < 0.05; **p < 0.01; ***p < 0.001). Specific Wald z statistics and two-sided *p* values are provided in Supplementary Data 1. No multiple-comparison adjustment was applied.

**Fig. 4:**
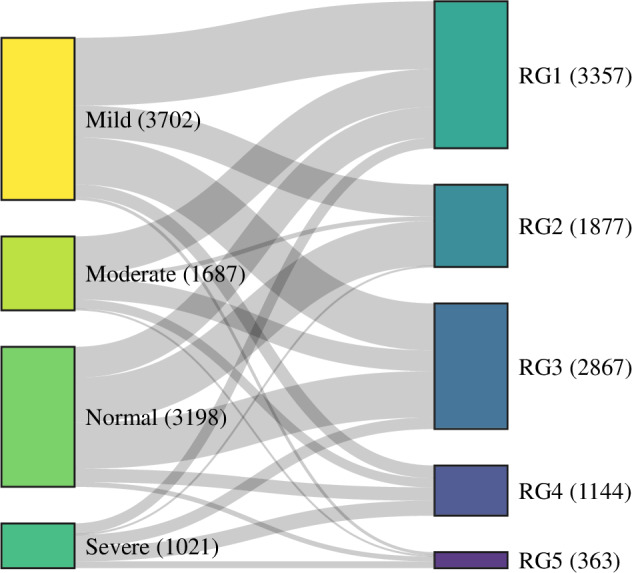
Relationship between AHI severity categories and embedding-derived risk groups. Sankey diagram showing the distribution of patients across conventional AHI severity categories with embedding-derived risk groups. Patients with severe AHI were distributed across multiple risk groups, while the highest-risk group (RG5) included patients spanning all AHI severities. These findings demonstrate that the learned physiologic embeddings capture clinically meaningful subtypes that are not explained by conventional AHI alone.

**Fig. 5:**
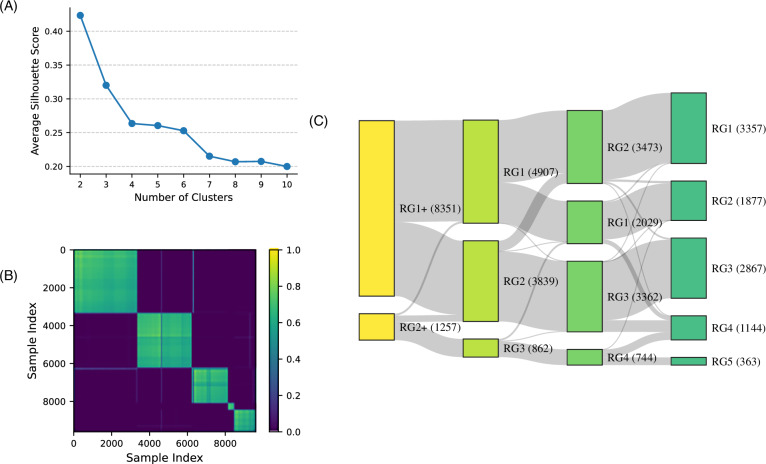
Stability of embedding-derived clustering solutions. **A** Silhouette scores for 2–10 clusters. **B** Consensus matrix for the five-cluster solution, showing stable block structure. **C** Sankey diagram mapping cluster assignments across 2–5 cluster solutions. Together, these analyses support five clusters as the most stable and clinically informative configuration.

**Fig. 6:**
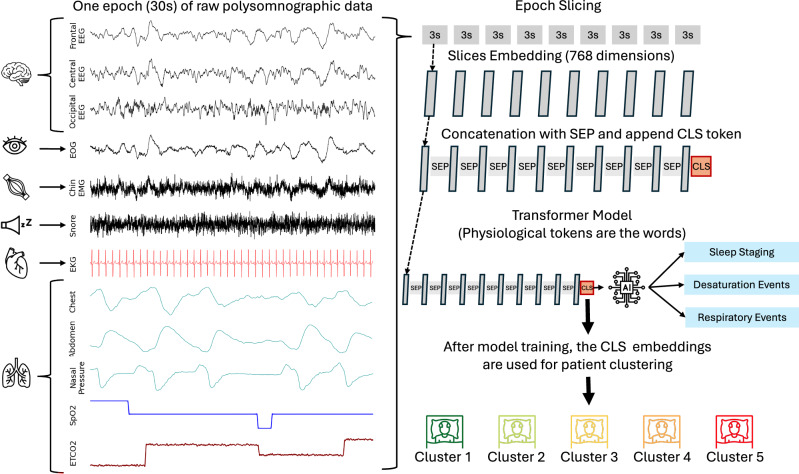
Tokenization framework for polysomnography (PSG) time-series foundation model. Architecture of the PSG foundation model illustrating the multimodal time-series tokenization strategy. Each 30-second PSG epoch was divided into 3-second multimodal patches spanning electrophysiologic, respiratory, and oxygenation signals, projected into a 768-dimensional embedding space, and concatenated with separator (SEP) tokens and a classification (CLS) token before processing by a transformer backbone. The CLS token summarizes global physiologic dynamics for supervised tasks, including sleep staging, respiratory event detection, and desaturation detection, and its learned embeddings are subsequently used for patient clustering. This tokenization framework enables multimodal and multiscale PSG time series to be represented using foundation-model techniques originally developed for text and forms the basis of the physiologic embeddings used in clustering and outcome prediction.

**Fig. 7:**
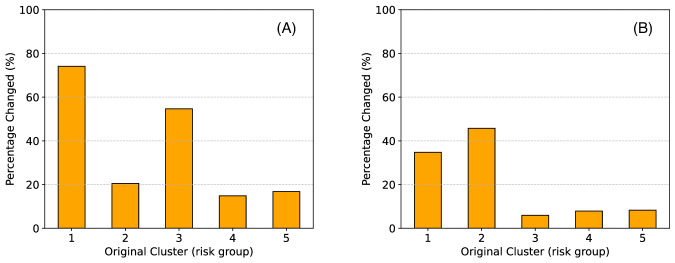
Sensitivity analyses of multimodal tokenization. Zeroing out EEG inputs during embedding generation led to marked increases in cluster reassignment across the original risk groups (**A**), while zeroing out ECG inputs produced a distinct reassignment profile (**B**). These results confirm that the learned embeddings integrate information across multiple physiologic systems and are not driven solely by respiratory signals. Figure 7 builds directly on the tokenization framework shown in Fig. 6.

We developed a foundation model for sleep that produced physiologic embeddings that stratified patients. These embeddings yielded five risk groups that showed strong, monotonic associations with incident cardiovascular, neurologic, and psychiatric outcomes (Fig. 1), corroborated by survival analyses in both the STARLIT-10K cohort and the independent SHHS cohort (Fig. 2). In contrast, standard AHI severity categories demonstrated no predictive value (Fig. 3), with embedding-derived groups revealing high-risk patients scattered across AHI strata (Fig. 4). These outcome-based results were supported by analyses demonstrating the stability of the five-cluster solution (Fig. 5), and by the reported time-series tokenization method (Fig. 6) and multimodal sensitivity testing (Fig. 7), which confirmed that embeddings captured physiologic information extending beyond respiratory signals alone.

### Foundation model development and validation

To create physiologic embeddings suitable for downstream clustering and outcome analysis, we developed and validated a transformer-based foundation model for PSG (Fig. 6). The model was trained simultaneously on sleep staging, respiratory events, and oxygen desaturations, enabling the generation of reusable physiologic high-dimensional representations (i.e., embeddings). The analytic workflow comprised three steps: representation learning from PSG signals, unsupervised stratification of patients, and evaluation of clinical outcomes. First, the transformer-based foundation model was trained on raw PSG time series using technologist annotations for staging and events, producing high-dimensional embeddings of sleep physiology. Next, these embeddings were used for unsupervised clustering to identify distinct patient subgroups. Finally, the clinical relevance of the subgroups was tested through longitudinal outcome analysis, including cardiovascular and neurological conditions as well as all-cause mortality.

We adapted a RoBERTa-based transformer for multimodal PSG by projecting time-series patches into a shared 768-dimensional embedding space and fine-tuning all weights for sleep staging, respiratory event, and desaturation tasks. Unlike prior approaches that froze pretrained weights, our adaptation refined the entire network to the characteristics of PSG waveforms and multi-modal time-series. The resulting model achieved strong classification performance across tasks, compared to prior studies^13^, demonstrating that it learned physiologically relevant features.

Model inputs included a comprehensive set of electroencephalograms (EEG), electro-oculograms (EOG), electromyograms (EMG), electrocardiograms (ECG), and ventilatory channels, providing a multimodal snapshot of sleep physiology. Specifically, the dataset included C4 (right central EEG), F4 (right frontal EEG), O2 (right occipital EEG), SpO_2_ (oxygen saturation measured via pulse oximetry), ECG (cardiac electrophysiologic signal), E1 and E2 (left and right eye EOG), chin EMG (submental muscle EMG), nasal pressure (airflow measurement via nasal pressure transducer), airflow (oronasal thermistry), chest and abdomen effort (inductance plethysmography for thoracoabdominal movement), snore (vibration sensor), and EtCO_2_ (end-tidal carbon dioxide level). Each 30-second PSG epoch was divided into ten non-overlapping 3-second segments, projected into the embedding space, and concatenated with separator tokens, producing ten tokens per channel.

A learnable CLS token condensed the multi-channel dynamics of each PSG into a compact representation while preserving physiologic richness for classification tasks. The CLS embedding was passed through task-specific linear layers for sleep staging, respiratory event detection, and desaturation detection, with cross-entropy–based losses ensuring balanced optimization across objectives.

### Model classification performance

Model classification performance was evaluated across three supervised tasks to confirm that embeddings captured physiologically relevant features. The tasks included (1) sleep stage recognition (non-rapid eye movement stages) across N1, N2, N3, and REM; (2) respiratory event detection (apneas and hypopneas, defined by 3% desaturation or arousal, or by 4% desaturation in line with insurance requirements); and (3) oxygen desaturation event detection.

For sleep staging, the model achieved high accuracy, with macro- and micro-averaged F1 scores of 0.75 and 0.86, respectively, consistent with prior benchmarks. Average precision (AP) was 0.62 (macro) and 0.76 (micro), confirming that the model reliably captured staging patterns. These metrics align with previously reported PSG-based classification studies^14^.

Performance for respiratory and oxygen desaturation was moderately lower, reflecting the inherent challenges of event labeling in 30-second epochs. Respiratory event detection yielded an F1 score of 0.65 and AP of 0.72, and oxygen desaturation detection yielded an F1 score of 0.59 and AP of 0.69. Because the labeling criterion required only five seconds of overlap with a manual annotation to mark an epoch positive, events spanning multiple epochs diluted per-epoch clarity, contributing to reduced scores. Importantly, these classification tasks are intermediate objectives designed to ensure that the model learns a physiologically relevant feature space, forming the basis for our main goal of generating embeddings used towards risk group clustering.

Together, these classification tasks served as intermediate objectives, ensuring the model learned a physiologic feature space suitable for clustering and outcome analyses. While not the study endpoint, these results demonstrate that the embeddings were clinically meaningful and robust enough to support downstream unsupervised analyses.

To assess whether clusters were driven primarily by respiratory signals, we performed modality sensitivity analyses. Zeroing out EEG or ECG inputs during embedding generation resulted in substantial misclassification of patients in risk groups RG1–RG3 (Fig. 7), demonstrating that non-respiratory physiologic channels contribute critically to cluster definition. This indicates that the model leverages multimodal PSG information beyond respiratory scoring to identify distinct sleep phenotypes.

### Cluster embeddings into risk groups

Clustering of physiologic embeddings revealed five stable and clinically distinct patient groups (Fig. 5), which we defined as risk groups (RG1–RG5). Embeddings from all PSGs that passed quality thresholds were analyzed using k-means with varying k-values, distance metrics, and sampling strategies (Supplementary Figs. 1–3). Silhouette score and consensus matrices identified the five-cluster solution, based on energy distance across all samples, as the most robust configuration (Supplementary Fig. 1). These clusters were subsequently validated through disease incidence and mortality analyses, enabling *a posteriori* labeling of risk groups. Results from Cox regression and propensity score matching further supported the stability and clinical relevance of these assignments (see Supplementary results).

The five-cluster solution provided the greatest granularity (Fig. 5A, B), ranging from low-risk groups with minimal abnormalities (RG1 and RG2) to a smaller, high-risk group (RG5) characterized by multiple comorbidities and PSG abnormalities consistent with severe sleep disruption. Intermediate groups (RG3 and RG4) exhibited distinct physiologic and clinical profiles that distinguished them from both the low- and high-risk extremes (Fig. 1). For comparison, a simpler two-cluster solution identified a high-risk subgroup (RG2^+^) that largely overlapped with RG5 (Fig. 5C). While this overlap is notable, the five-cluster configuration offered greater differentiation of patient trajectories and serves as the primary focus of subsequent analyses.

### Associations between risk groups and incidence of clinical outcomes

Across the five-cluster solution, disease incidence increased progressively from RG1 to RG5, with the highest risks consistently observed in RG5 (Fig. 1). Baseline multivariable logistic associations between risk groups and prevalent comorbidities showed a similar graded pattern (Supplementary Table 4). Full model coefficients, Wald *z* statistics, and specific two-sided *p* values for these baseline logistic analyses are provided in Supplementary Data 5. Associations remained robust after propensity-score matching and trimming for outliers, indicating that clustering-derived groups captured physiologic differences beyond conventional AHI classification (Supplementary Fig. 4, Supplementary Table 2).

RG2 showed little evidence of excess risk compared to RG1 (Fig. 1). Most associations were close to null, and some conditions occurred less frequently in RG2, including hyperlipidemia (HR = 0.75, 95% CI = 0.63–0.89, *p* < 0.01) and obesity (HR = 0.79, 95% CI = 0.68–0.93, *p* < 0.01).

RG3 displayed a moderate but broad increase in disease risk. Associations were elevated for type 2 diabetes (HR = 1.34), hyperlipidemia (HR = 1.30), major adverse cardiovascular events (MACE; HR = 1.44), ischemic heart disease (HR = 1.94), myocardial infarction (HR = 1.42), stroke (HR = 1.44), and mood disorders (HR = 1.46), all statistically significant.

RG4 showed further increases, particularly for type 2 diabetes (HR = 1.27), MACE (HR = 1.33), coronary artery disease (HR = 1.32), atrial fibrillation (HR = 1.40), cognitive impairment (HR = 1.42), and gastroesophageal reflux disease (GERD; HR = 1.32).

RG5 consistently exhibited the strongest associations across outcomes, including MACE (HR = 1.64), heart failure (HR = 1.65), myocardial infarction (HR = 1.84), atrial fibrillation (HR = 2.23), cognitive impairment (HR = 1.93), and epilepsy (HR = 2.40). These findings highlight an increased risk of serious cardiovascular and neurological conditions in this subgroup.

Multiple propensity score-matched analyses confirmed the robustness of these results. Disease-free survival declined progressively across groups (Supplementary Fig. 4), from 80.6% in RG1 to 67.3% in RG5 for MACE (*p* < 0.001), and from 84.3% in RG1 to 75.2% in RG5 for cognitive impairment (*p* < 0.01). Supplementary Table 2 presents propensity score-matched HRs, which preserved the same overall trend: modest elevation in RG3, intermediate elevation in RG4, and the strongest associations in RG5. Supplementary Data 1—2 provide extended results for each incident disease.

To test whether these risk groups reflect more than standard AHI severity, we directly compared the embedding-derived clusters to AHI categories. In fully adjusted Cox models that included AHI as a covariate, cluster membership remained strongly associated with all-cause mortality (e.g., RG5: HR = 2.38, *p* = 1.2 × 10⁻⁷) and with cardiovascular and neurological outcomes (Table 1). A Sankey diagram (Fig. 4) further showed that the five clusters integrate patients across multiple AHI levels in non-linear patterns, confirming that the physiologic subtypes captured by the model cannot be explained by AHI severity alone.

**Table 1:** Hazard ratios of all-cause mortality among patients from different risk groups

Model	No samples	No events	RG2	RG3	RG4	RG5
1	7043	544	0.92 (0.72–1.17)	1.69 (1.29–2.23)***	3.11 (2.46–3.92)***	4.83 (3.60–6.50)***
2	7043	544	1.54 (1.20–1.98)***	1.65 (1.25–2.16)***	1.84 (1.45–2.33)***	2.73 (2.02–3.69)***
3	7043	544	1.47 (1.14–1.88)**	1.52 (1.16–2.00)**	1.67 (1.31–2.11)***	2.16 (1.59–2.95)***
4	7043	544	1.43 (1.11–1.84)**	1.54 (1.17–2.03)**	1.75 (1.37–2.23)***	2.38 (1.73–3.28)***
5	3700	299	1.32 (0.95–1.82)	1.66 (1.14–2.40)**	1.78 (1.28–2.47)***	1.76 (1.12–2.78)*
6	1569	523	1.53 (1.15–2.04)**	1.47 (1.08–2.01)*	1.67 (1.30–2.15)***	1.75 (1.24–2.49)**

Model 1: without adjustment; 2: adjusted for age, gender and body mass index (BMI); 3: adjusted for age, gender, BMI and comorbidities (hypertension, diabetes type 2, heart failure, atrial fibrillation, coronary artery disease, hyperlipidemia); 4: adjusted for age, gender, *BMI* comorbidities and AHI; 5: adjusted for age, gender, BMI, comorbidities and excluding patients for which PAP therapy was prescribed; 6: adjusted for age, gender, BMI and comorbidities excluding patients less than 55 years of age. Hazard ratios are from Cox proportional hazards models with risk group 1 as the reference group and are reported with 95% confidence intervals. Asterisks denote two-sided Wald-test *p* values (*p < 0.05; **p < 0.01; ***p < 0.001). Specific Wald *z* statistics and two-sided *p* values for all Cox models shown in Table 1 are provided in Supplementary Data 1.

Risk groups demonstrated strong and independent stratification of all-cause mortality, with higher-risk groups showing progressively reduced survival compared to RG1 (Fig. 2A). Over a mean follow-up of 4.9 ± 3.3 years, mortality increased monotonically from RG2 through RG5. Based on this, its large sample size and consistently low-risk profile, we chose RG1 as the reference group providing a stable baseline for comparisons.

Cox regression models adjusted for demographics, BMI, comorbidities, and AHI confirmed significantly elevated mortality hazards for RG2–RG5, independent of traditional AHI categories. In the fully adjusted model (Table 1, Model 4), hazard ratios (HRs) remained significant: RG2 = 1.43 (*p* = 0.0052), RG3 = 1.54 (*p* = 0.0020), RG4 = 1.75 (*p* = 5.82×10⁻⁶), and RG5 = 2.38 (*p* = 1.20×10⁻⁷). This ordinal pattern persisted after propensity-score matching and trimming, demonstrating that the risk groups captured prognostic signal beyond conventional covariates.

By contrast, AHI-defined categories of mild, moderate, and severe sleep apnea showed no significant association with mortality (Fig. 3). A Cox model substituting AHI categories for risk groups, using the same covariates, showed no increase in mortality relative to AHI < 5. For example, after excluding patients with positive airway pressure (PAP) prescriptions (defined as the presence of an order for a positive airway pressure device in the electronic health record), hazard ratios for mild, moderate, and severe AHI remained non-significant (HR = 0.79, HR = 0.70, HR = 1.05, respectively).

Findings were robust in models excluding PAP-treated patients and those under 55 years, reinforcing that mortality risk is not explained by treatment exposure or age distribution. Excluding PAP prescriptions (Table 1, Model 5) or restricting to ≥55 years (Table 1, Model 6) yielded materially similar HRs, and matched analyses showed progressively decreased disease-free survival across groups. Elevated hazards for RG2–RG5 persisted under these restrictions, and results also remained significant after adjustment for AHI, demonstrating that the prognostic signal captured by the embedding-derived risk groups was independent of traditional severity thresholds.

### Predicting clusters using standard PSG metrics

Embedding-derived risk groups were also compared to conventional PSG variables to assess whether standard measures could approximate cluster membership (Supplementary Table 3). Features considered included AHI, arousal index, total sleep time, sleep stage percentages, oxygen saturation, and a quantitative measure of Spectral Sleep Fragmentation (SSF, see Methods), which quantifies the proportion of rapid stage transitions (<10 min) in the hypnogram power spectrum.

For the two-cluster solution, classification performance was high (Supplementary Table 3). Using SSF alone, prediction accuracy exceeded 90%, and incorporating the top five PSG features increased the accuracy to 94%.

In contrast, prediction accuracy deteriorated substantially for the three-, four-, and five-cluster solutions (Supplementary Table 3). Even when multiple features were combined, conventional PSG metrics could not reliably reproduce the embedding-derived stratification. These findings indicate that while simple separations can be captured by familiar variables, the richer, multidimensional sleep profiles identified by embeddings cannot be fully reduced to traditional PSG measures.

### External validation of embedding-derived sleep profiles

To externally validate our risk stratification pipeline, we applied it to the independent Sleep Heart Health Study (SHHS) cohort^15,16^, a prospective study with detailed PSG data. Despite differences in PSG channel availability (e.g., limited to nasal pressure, thermistor, and thoracic/abdominal bands in SHHS versus multichannel clinical recordings) and signal sampling rates, our pipeline yielded consistent associations with clinical outcomes, underscoring its robustness across diverse datasets.

Figure 2 presents Kaplan-Meier survival curves for all-cause mortality (A and B) and incident heart failure (C and D) stratified by risk groups (RG1–RG5), with panels A and C from our propensity score-adjusted clinical cohort (corrected for demographics to approximate a controlled prospective design, as detailed in Methods) and B and D from SHHS. In both cohorts, analyses were further adjusted for age and gender following the approach in prior SHHS studies^17^.

In our clinical cohort, higher risk groups showed significantly reduced survival for all-cause mortality (Fig. 2A; log-rank ${\chi }^{2}(4)$ = 67.02, *p* = 6.25×10^−14^ for RG3–RG5 vs. RG1), with age/gender-adjusted Cox proportional hazards ratios (HRs) demonstrating a dose-response relationship: HR = 1.51 (95% CI = 1.17–1.96, *p* = 0.0016) for RG3, HR = 1.64 (95% CI = 1.26–2.15, *p* = 2.8×10⁻⁴) for RG4, and HR = 2.71 (95% CI = 1.93–3.81, *p* = 8.5×10⁻⁹) for RG5, all versus RG1. Similarly, for heart failure (Fig. 2C), RG5 was associated with significantly worse outcomes (log-rank ${\chi }^{2}\left(4\right)$ = 23.85, *p* = 8.55×10^−5^ vs. RG1; adjusted HR = 1.67, 95% CI = 1.12–2.48, *p* = 0.011).

External validation in SHHS replicated these patterns. For all-cause mortality (Fig. 2B), RG4 and RG5 showed significant associations (log-rank ${\chi }^{2}(4)$ = 121.08, *p* = 3.14×10^−25^ vs. RG1), with adjusted HRs of 1.31 (95% CI = 1.07–1.62, *p* = 0.009) for RG4 and 1.58 (95% CI = 1.07–2.32, *p* = 0.021) for RG5. For incident heart failure (Fig. 2D), RG5 again emerged as a key predictor (log-rank ${\chi }^{2}(4)$ = 31.75, *p* = 2.15×10^−6^ vs. RG1; adjusted HR = 2.13, 95% CI = 1.11–4.09, *p* = 0.023).

These findings contrast with original SHHS analyses using AHI, which identified associations with all-cause mortality primarily in men aged 40–70 years with severe SDB (AHI ≥ 30; adjusted HR = 1.46 overall, 2.09 in this subgroup), with no significant links in women^18^. Similarly, AHI-based assessments linked incident heart failure only to severe OSA in men (AHI ≥ 30; adjusted HR = 1.58), but not in women^17^. Our method, however, demonstrated consistent prognostic value across cohorts without such restrictions to severe cases or specific demographics, highlighting its enhanced sensitivity for risk detection in heterogeneous populations.

## Discussion

We developed a sleep foundation model that leverages multimodal PSG signals and EMRs to identify latent physiologic patterns predictive of long-term clinical outcomes. By embedding continuous time-series signals into high-dimensional representations and clustering these embeddings, we identified distinct patient risk groups that stratified cardiovascular, neurological, and survival outcomes more effectively than conventional metrics. Notably, AHI categories failed to predict mortality, whereas embedding-derived groups revealed strong, graded associations with major adverse cardiovascular events, atrial fibrillation, cognitive impairment, and epilepsy. These findings demonstrate that foundation models can extract prognostic biomarkers directly from routine PSG, moving beyond traditional indices to provide clinically meaningful risk stratification.

A central finding of this study is the demonstration that PSG embeddings provide disease-specific prognostic information that cannot be otherwise obtained by AHI alone. For decades, sleep-disordered breathing severity has been defined by AHI, a frequency-based metric of airway obstruction events. Yet, AHI showed no association with mortality in our cohort, replicating a consistent limitation across prior epidemiologic studies. In contrast, our risk groups separated patients along trajectories of incident cardiovascular and neurological disease and overall survival, even after adjusting for demographics, comorbidities, and AHI itself. This confirms that physiologic embeddings capture broader sleep pathophysiology, incorporating neural, cardiac, and ventilatory signals that together explain outcomes beyond airway obstruction alone. Sensitivity analyses zeroing out EEG and ECG channels further underscored the importance of these modalities, demonstrating that the prognostic ability of the model is not solely respiratory-driven.

Our approach addresses an important need within sleep medicine. The American Thoracic Society (ATS) has explicitly prioritized the development of scalable, objective measures that go beyond AHI to predict cardiovascular and neurological outcomes^19^. Current metrics, such as hypoxic burden and heart rate response, while promising, each capture only isolated aspects of physiology. Similarly, prior clustering studies using aggregate PSG indices identified broad phenotypes but were constrained by reliance on coarse, technologist-scored measures^20,21^. By contrast, foundation models learn directly from raw multimodal data to generate general-purpose embeddings without hand-crafted features. In doing so, our work directly responds to ATS priorities by providing a scalable, validated framework that uncovers hidden physiologic signatures with clear clinical implications.

We also demonstrate the robustness of our approach through external validation. Applying the two-cluster separation based on SSF to the population-based SHHS reproduced associations with mortality and heart failure in both men and women. This contrasts with the original SHHS analysis, which identified an association only between severe AHI and heart failure in men. The consistency of our embedding-derived risk groups across cohorts from independent institutions, each with its own protocols for PSG (a clinical referral population and a community-based cohort with lower-resolution PSG), underscores the generalizability of our results. Importantly, SHHS participants assigned to the high-risk group exhibited markedly short total sleep times, mirroring the marked physiologic disruption observed in our highest-risk cluster, further supporting biological plausibility and clinical relevance.

Among the clustering solutions tested, the five-group framework captured nuanced heterogeneity in patient trajectories, while the simpler two-group solution offered practical advantages for external application. The two-cluster classifier, driven primarily by our SSF metric, achieved >90% accuracy and generalized robustly across cohorts. This suggests that while embeddings are essential for discovering fine-grained risk groups, simplified metrics can support translation to diverse settings, particularly when standard, more basic PSG data are available. Together, these approaches illustrate a continuum: Complex embeddings provide granularity and interpretability, while future more streamlined surrogates enable scalability.

Our study has several important strengths. First, we leveraged STARLIT-10K, a large and demographically diverse clinical cohort enriched for minority representation, with detailed longitudinal EMR follow-up extending over 14.5 years. This scale and diversity enabled precise estimation of long-term associations across a broad range of outcomes, enhancing generalizability and clinical relevance. Second, by integrating multimodal PSG signals with EMR data, we combined physiologic richness with detailed phenotyping, allowing more comprehensive risk stratification than either source alone. Third, our use of propensity score matching and sensitivity analyses minimized confounding and ensured that observed associations reflect underlying physiology rather than demographic or treatment differences.

Nevertheless, several limitations should be acknowledged. The foundation model is trained using supervised objectives (sleep stages, respiratory events, and oxygen desaturations), and therefore the learned embedding is necessarily influenced by these tasks. We do not claim that the representation is label independent. Rather, the use of multiple, partially independent supervision signals is intended to reduce reliance on any single annotation system and encourage learning of shared physiological structure. Importantly, conventional PSG features derived from these same annotations are insufficient to reproduce the embedding-derived cluster structure, indicating that the learned representation captures physiological information beyond standard task-defined metrics. Future work will explore self-supervised and disease-targeted objectives to further reduce dependence on technician-defined labels. Objective adherence data for positive airway pressure (PAP) therapy were unavailable, which may have influenced outcomes. While we excluded patients with PAP prescriptions in sensitivity analyses and found consistent results, incomplete treatment data could bias associations toward the null. Similarly, medication use was not systematically recorded, and residual confounding cannot be excluded. Reliance on EHR-based ICD-10 diagnostic codes may introduce misclassification due to variable coding practices and incomplete capture of clinical detail^22^, though our adjudication pipeline and clinician review achieved >90% agreement. The retrospective design limits causal inference, underscoring the need for future prospective studies. Finally, although external validation in SHHS supports generalizability, replication in additional cohorts and prospective clinical trials will be critical before implementation and deployment in healthcare settings.

The clinical implications of this work are significant. Embedding-derived risk groups identify subpopulations at elevated risk for mortality and disease who would be missed by AHI-based classification. Patients in the highest-risk cluster (RG5) may warrant targeted interventions that extend beyond treatment of sleep-disordered breathing, such as cardiovascular monitoring or early neurologic evaluation. Conversely, patients in low-risk groups could avoid unnecessary interventions, supporting more personalized care pathways. At a population level, embedding-informed stratification could refine referral patterns, optimize allocation of resources, and inform trial design by enriching for high-risk participants most likely to benefit from intervention.

Our findings also open new directions for future research inquiries. Patient-reported outcomes and symptom measures were not included here but remain central to sleep medicine; future work integrating subjective data with embeddings may yield more informative phenotypes. Embedding-derived groups could also inform mechanistic studies, guiding exploration of how neural, respiratory, and cardiovascular signals causally interact to drive morbidity. Importantly, prospective trials are needed to test whether embedding-based risk stratification improves patient outcomes when integrated into clinical workflows. Implementation science approaches will be key to translating this framework into clinically viable solutions.

In conclusion, we present a foundation model for sleep that learns physiologic embeddings from routine PSG and identifies risk groups predictive of cardiovascular, neurological, and survival outcomes. By moving beyond AHI, this work addresses long-standing limitations in sleep medicine and aligns directly with current field priorities to identify scalable, physiologically grounded biomarkers. The model generalizes across clinical and population-based cohorts, demonstrating both robustness and clinical relevance. Together, these results establish an AI foundation model for embedding-based risk stratification to inform precision sleep medicine, guiding monitoring, referral, and intervention in ways that traditional approaches cannot.

## Methods

### Data collection

The dataset derives from a retrospective clinical cohort study approved by the Cleveland Clinic Institutional Review Board (IRB#23-409) in 2023 under expedited review with a waiver of Informed Consent given the data were de-identified, resulting in minimal risk to participants. As such, participants did not receive compensation. The STARLIT-10K (Sleep Signals, Testing, and Reports Linked to Patient Traits) cohort originally comprised 10,000 in-laboratory PSG studies from 9661 patients linked to EMRs, forming a demographically diverse resource for investigating long-term sleep-related health outcomes. Following data quality control and preprocessing, 9608 PSGs from 9297 unique patients were retained and used for clustering analyses. Gender was obtained from the demographic gender data field of the STARLIT-10K clinical dataset, which reflects patient-reported gender identity. A small proportion of participants (approximately 0.2%) had a gender designation that differed from sex assigned at birth. Accordingly, we use the term gender throughout the manuscript, consistent with Nature Portfolio guidance. Sleep studies were conducted at the Cleveland Clinic between January 2012 and December 2022 using Nihon Kohden equipment and Polysmith software to record physiologic signals, stage sleep, generate epoch-by-epoch annotations, and produce finalized reports by board-certified sleep physicians. The first 1000 PSGs were selected randomly, and the remaining 9000 PSGs were sampled to ensure age diversity, temporal representation, and enriched inclusion of minority participants across the cohort.

Clinical outcomes and longitudinal data were derived from linked EMRs and mortality registries, providing comprehensive follow-up for each participant. Polysomnographic metrics were extracted from the structured HTML format of the final sleep study reports, including conventional sleep variables and derived physiologic measures defined in Supplementary Table 1. Each PSG metric is contained within a predefined identified tag, allowing for deterministic HTML parsing. Study outcomes were identified from a predefined vocabulary of diagnoses after medical consensus and their corresponding ICD codes in the EMR. All-cause mortality was ascertained by integrating EMR updates with the Ohio Death Index and the Social Security Death Index.

The cohort was characterized by extended longitudinal coverage before and after PSG testing. Patients had a median of 9.4 years of medical history prior to PSG (IQR: 4.6, 13.6) and 4.4 years of follow-up after testing (IQR: 2.0, 5.7), yielding a total median observation window of 15.1 years (IQR: 9.4, 19.0) and a mean observation period of 14.5 ± 7.1 years. A more detailed description of the STARLIT-10 K dataset and its multi-institutional collaborations is provided in Bilal et al. ^23^ and Supplementary Data 4.

### Data preprocessing

PSG signals from multiple physiologic modalities were preprocessed using standardized procedures to ensure consistency across recordings while retaining physiologic integrity. Each PSG included data from EEG, EOG, EMG, ECG, respiratory airflow, thoracic and abdominal movement, EtCO_2_, snoring, and SpO_2_ sensors. All signals were resampled to 128 Hz and preprocessed following the approach described in Brink-Kjaer et al. ^24^ to remove artifacts and harmonize recordings across studies.

Spatial filtering was implemented using 16th-order elliptic infinite impulse response (IIR) filters with a maximum passband ripple of 1 dB, and a minimum stopband attenuation of 40 dB. Cut-off frequencies were modality-specific: EEG and EOG (0.3–45 Hz, band-pass), EMG (10 Hz, high-pass), ECG (0.3 Hz, high-pass), nasal pressure (0.1 Hz, high-pass), airflow and plethysmography belts (0.1–15 Hz, band-pass), and no filtering for SpO_2_. Filters were applied forward and backward to avoid phase distortion. Finally, signal amplitudes were normalized such that −1 and 1 corresponded to the 5th and 95th percentiles for all channels except SpO_2_, which was normalized to −1 and 1 corresponding to 60% and 100% saturation.

### Foundation model training and embedding generation

We trained the PSG foundation model on 9203 studies using a multi-task objective designed to capture physiologic features across multiple domains. The foundation model was designed to address three tasks: sleep stage classification, respiratory event detection (e.g., apneas and hypopneas), and oxygen desaturation identification. The joint training objective is used as a physiological constraint rather than as a final endpoint, ensuring that embeddings remain grounded in well-defined PSG signals while avoiding optimization toward any single task-specific feature set. Of the available PSGs, 30 were reserved for validation and 500 for testing, with inclusion criteria detailed in Supplementary methods.

Model optimization employed task-specific loss functions within a transformer-based architecture to ensure balanced performance across objectives. Training used the Adam optimizer^25^ with a learning rate of 1 × 10^−5^, a batch size of 500, and 50 training epochs. Losses were calculated using cross-entropy for sleep staging and binary cross-entropy for desaturation and respiratory event detection, with the overall loss defined as the average of the task-specific losses. The architecture followed the RoBERTa-base design with 12 transformer layers, a 768-dimension representation, and approximately 126 million trainable parameters.

Physiologic embeddings were derived by aggregating CLS token representations from sequential 30-second epochs into study-level matrices. For each PSG, epoch- CLS tokens were stacked to generate a two-dimensional embedding matrix with 768 rows and columns corresponding to the number of 30-second epochs in the recording. These matrices provided fixed representations for subsequent clustering analysis. The model was implemented in PyTorch using the HuggingFace model library, and training was performed on two NVIDIA A40 GPUs with 46 GB memory each.

### Clustering method

To enable clustering of variable-length PSG embeddings, we transformed each study into a fixed-size feature vector suitable for k-means analysis. After model training, the CLS token for each 30-second epoch was stacked to create a two-dimensional embedding matrix with 768 rows and a variable number of columns reflecting PSG duration. Because study length differed across patients, k-means clustering could not be applied directly. To address this, we calculated pairwise distances between each embedding and all other embeddings by aligning rows (time dimension) and averaging across the feature dimension. This process produced a fixed-length distance vector for each study, which was then used as input for clustering.

We evaluated distance metrics capable of comparing embeddings of different lengths, focusing on energy distance^26^ and Earth mover’s distance^27^, both well-suited for distributions with unequal sample sizes. To assess the effect of sample size, we performed analysis using all available PSGs as well as projections restricted to the test set.

Clustering performance was evaluated using silhouette scores and consensus matrices to quantify cohesion, separation, and stability. The silhouette score quantified the cohesion and separation of clusters, with higher scores indicating better-defined groups. The consensus matrix was generated through subsampling, where 80% of samples and features were randomly selected in 100 iterations. For each pair of samples, the matrix recorded the proportion of iterations in which the samples were assigned to the same cluster. To facilitate interpretation, the consensus matrix was ordered using hierarchical clustering with the Ward criterion^28^, grouping samples based on their co-clustering frequency to visually highlight stable cluster structures.

Consensus analysis demonstrated that clustering was stable up to five groups, with structure deteriorating beyond this point (Supplementary Fig. 1). Energy distance applied to all samples yielded a block-diagonal consensus structure and consistently high silhouette scores, indicating robust group formation. Sensitivity analyses confirmed the robustness of the five-cluster solution across alternative metrics and sample subsets (Supplementary Figs. 2, 3). Additional comparisons across three independent five-cluster solutions further supported stability (Supplementary Fig. 5). Based on these results, the five-cluster configuration using energy distance was selected for subsequent analyses.

### Statistical analyses

We first compared baseline demographic and clinical characteristics across risk groups to evaluate group differences prior to outcome analyses. Categorical variables were assessed using chi-square tests with Bonferroni corrections for pairwise comparisons on significant results (*p* < 0.05). Continuous variables were analyzed using Welch’s ANOVA, followed by pairwise *t*-tests with Bonferroni correction when significant. Logistic regression was used to examine associations between risk groups and baseline medical history, adjusting for age, BMI, gender, and comorbidities (Supplementary Data 3).

Time-to-event outcomes were modeled using Cox proportional hazards regression, with survival defined from the date of baseline PSG to the date of death or censoring at last follow-up. Models included demographic variables (age, gender, BMI), comorbidities (Supplementary Data 3), AHI, and clustered risk groups. Only patients with at least five years of prior medical history and no prior occurrences of the outcome were included. Proportional hazards assumptions were tested and satisfied for all models.

Robustness of associations was tested using propensity score–matched Cox regression and Kaplan–Meier survival analyses. Propensity scores were estimated using multinomial logistic regression based on age, BMI, gender, comorbidities, and length of medical history available before sleep testing. Stabilized inverse propensity^29^ weights were applied and trimmed at the 5th and 95th percentiles to reduce bias from extreme values. Kaplan-Meier curves were generated to estimate disease-free survival at 2, 4, and 6 years, with log-rank tests comparing survival rates across groups.

Generalizability was assessed by applying the full embedding and clustering pipeline to SHHS, a publicly available, population-based cohort of >6000 participants (https://sleepdata.org/datasets/shhs). PSGs were resampled to match STARLIT-10K resolution and preprocessed using the same procedures. Embeddings were generated using the pretrained foundation model without modification, and fixed k-means centroids from STARLIT-10K were used for cluster assignment. Outcomes included all-cause mortality and incident congestive heart failure, harmonized with STARLIT-10K endpoints. Survival analyses were conducted as in STARLIT-10K, adjusting for age, gender, and BMI, enabling direct comparison of risk group performance across independent clinical and population-based cohorts.

### Spectral sleep fragmentation metric

We defined a quantitative SSF measure based on the frequency spectrum of stage transitions in the hypnogram. The hypnogram sequence $h\left(t\right)$ was first converted into a binary transition signal $T\left(t\right)$, where $T\left(t\right)=1$ indicated a stage change between consecutive 30-second epochs and $T\left(t\right)=0$ otherwise. A power spectral density (PSD) estimate of $T\left(t\right)$ was then computed, and the SSF score was defined as the normalized spectral power corresponding to transition frequencies faster than 10 minutes:

1 $${{\rm{SSF}}}=\frac{{\int }_{f\ge 1/600}P\left(f\right){df}}{{\int }_{f\ge 0}P\left(f\right){df}}$$

where P(f) is the PSD of T(t), and 1/600 Hz corresponds to transitions occurring more frequently than once every 600 s (10 min). A higher SSF value reflects a greater proportion of rapid transitions between sleep stages, consistent with more fragmented sleep architecture. Although Spectral Sleep Fragmentation (SSF) is derived from EEG dynamics related to sleep fragmentation, it is not explicitly optimized during training and cannot be reduced to apnea burden, desaturation frequency, or categorical sleep stage labels.

### Clinical outcome extraction

Clinical diagnoses and outcomes were derived using a natural language processing (NLP) pipeline developed and validated within STARLIT-10K, using the curated disease vocabulary listed in Supplementary Data 6. Structured fields (ICD-10 codes, medication orders) were combined with unstructured clinical text using a rules-based NLP system supplemented by dictionary lookups. Key outcomes included MACE, ischemic heart disease, stroke, heart failure, atrial fibrillation, epilepsy, cognitive impairment, and mood disorders.

To ensure reliability, outcomes were adjudicated by cross-referencing ICD codes, problem lists, and clinical notes. A random 5% sample was manually reviewed by two clinicians, achieving >90% agreement for outcome classification. Only adjudicated outcomes were retained for primary analyses.

### Missing data and imputation

Missing data were addressed using imputation strategies tailored to the extent of missingness. Variables with <5% missingness (e.g., BMI, smoking status) were imputed by mean or mode substitution, while variables with >5% missingness (e.g., comorbidity indicators) were multiply imputed using chained equations. Imputation models incorporated age, gender, BMI, and comorbidities to preserve relevant associations.

Robustness was confirmed by comparing hazard ratios from complete-case analyses with those obtained from imputed datasets, which showed consistent results in both magnitude and direction. This approach ensured that missing data did not introduce systematic bias into the associations between risk groups and clinical outcomes.

### Reporting summary

Further information on research design is available in the Nature Portfolio Reporting Summary linked to this article.

## Supplementary information

Supplementary Information

Description Of Additional Supplementary File

Supplementary Data 1

Supplementary Data 2

Supplementary Data 3

Supplementary Data 4

Supplementary Data 5

Supplementary Data 6

Reporting summary

Transparent Peer Review file

## Data Availability

The data that support the findings of this study are derived from two sources. Data from the Sleep Heart Health Study (SHHS) is promptly available for reproducible purposes through SleepData.org, subject to registration and approval under the repository’s data access policies. The Cleveland Clinic dataset was used under license for this study and is not publicly available due to institutional and ethical restrictions imposed by the Cleveland Clinic Institutional Review Board (IRB), which prohibits sharing data outside the approved study team. Access to these data requires submission of a new application for IRB approval at the Cleveland Clinic Foundation, along with execution of a data use agreement. Requests for access may be made by qualified researchers for purposes consistent with the original study objectives and ethical approvals. Requests should be directed to Dr. Matheus Araujo at limadim@ccf.org and should include details regarding the proposed research purpose. An initial response is typically provided within one week from Dr. Araujo. However, all requests must be reviewed by the Cleveland Clinic IRB and are subject to institutional approval, which typically requires 12-15 weeks. If approved, access will be granted for a limited period in accordance with the terms of the data use agreement.

## Code availability

The methodological workflow, model architecture, tokenization strategy, multitask training framework, and downstream analytical pipeline are described in detail in the Methods and illustrated in Fig. 6, providing sufficient information for independent reconstruction of the principal analytical approach. The original production-grade code used for foundation model training and deployment was developed within the IBM Research and Cleveland Clinic Discovery Accelerator and is not available due to institutional intellectual-property controls and legacy infrastructure dependencies. Processed analysis scripts used to reproduce the principal statistical analyses and main figures are available from the corresponding author, Jeffrey L. Rogers (jeffrey.rogers@yale.edu), for reproducibility-focused academic research within 30 days of request.
