# Supplementary material for: A foundation model for sleep-based risk stratification and clinical outcomes
Source: Nat Commun. 2026 Aug 3;17:7603. doi: 10.1038/s41467-026-75326-9 (PMC13434717; doi:10.1038/s41467-026-75326-9)
Supplement: Supplementary file 1 [file 41467_2026_75326_MOESM10_ESM.pdf]

nature portfolio

Peer Review File

A Foundation Model for Sleep-Based Risk Stratification and Clinical Outcomes

Corresponding Author: Dr Jeffrey Rogers

This file contains all reviewer reports in order by version, followed by all author rebuttals in order by version.

Attachments originally included by the reviewers as part of their assessment can be found at the end of this file.

Version 0:

Reviewer comments:

Reviewer #1

(Remarks to the Author)
see attached document for our review

Reviewer #2

(Remarks to the Author)
I co-reviewed this manuscript with one of the reviewers who provided the listed reports. This is part of the Nature Communications initiative to facilitate training in peer review and to provide appropriate recognition for Early Career Researchers who co-review manuscripts.

Reviewer #3

(Remarks to the Author)
This paper proposes a novel methodology for inferring polysomnography (PSG)-derived clusters at risk of clinical outcomes. This methodology is composed of two main steps:
(i) A Transformer model (RoBERTa model) that generates data-driven representations of latent physiological patterns. The pretrained RoBERTa model is fine-tuned with 30-s epochs of raw PSG channels (C4, F4, =2-M2, SpO2, ECG, E1, E2, chin EMG, nasal and thermistor airflow, chest and abdomen effort, snore, and etco2) to jointly detect sleep stages, oxygen desaturations events, and respiratory events (apnea/hypopnea). This model has a shared RoBERTa-based transformer backbone and task-specific linear layers for each task. The CLS tokens at the output of the backbone serve as the data-driven representations for each sleep study.
(ii) A clustering method that provides subpopulations with unique health characteristics. Taking as input a two-dimensional embedding matrix (stacked 30-s epoch representations of the CLS tokens) from each sleep study, this approach first projects it into a fixed-size feature vector using pairwise distances between its embedding and and the embeddings of all other simples. These feature vectors were then used as input for k-means clustering, returning a robust clustering approach for up to five clusters.
Although not higher than state-of-the-art (SOTA) studies, the Transformer model provided an acceptable performance for the three classification tasks. Conversely, the proposed methodology derived in clinically informative risk groups (i.e., clusters), which showed clear incremental risk associations with many diseases (cardiovascular, neurological, and others), including survival (the highest risk group: (unadjusted hazard ratio [HR] 4.83, 95% confidence interval [CI] 3.60–6.50, p<0.001) in a private database of nearly 10000 PSGs. Authors also provided an external validation of the 2-cluster approach in the Sleep Heart Health Study database (https://sleepdata.org/datasets/shhs), obtaining similar associations of the high-risk group with mortality and heart failure, in both sexes. However, several issues need to be addressed:
1. There are some statements without a single reference. For example, only one reference is provided within the first two paragraphs of the introduction.
2. Figure 1 should graphically include all the PSG channels used.
3. ¿Why do authors choose oxygen desaturations and not arousals or spindles detection? Further explanations should be provided.
4. Authors perform a multi-task (sleep stages, oxygen desaturations, and respiratory events) model. Why do author shave

choose it over single-task models (i.e., separate models and clusters for each task)? Further explanations should be provided to better highlight the advantages of multi-task learning. I leave as optional/future task to perform separate clustering approaches for three single-task models. This could help to support the suitability of multi-task learning for finding better clusters.
5. Why do authors only use artifact-free PSGs? DL methods are able to deal with artifacts.
6. With respect to model training: (i) why do authors stop it at epoch 50 rather than using early stopping? (i) why do authors use so few validation and test subjects? Further explanations should be provided.
7. I would include a figure to fully explain the clustering approach in a graphical way. This figure could be included in the main manuscript or as a supplementary material/file, and it should describe every step with input-output dimensions.
8. What is the hardware equipment used to train the models? This can help readers know the resources need to reproduce the proposed methodology
9. What is the software used to train the Transformer model? At least, authors should include the code of the foundation model training and embedding generation, as well as the clustering method. In this way, authors could reproduce the proposed methodology using other datasets such as SHHS.
10. At the end of "Cluster Embeddings into Risk Groups" authors should indicate that a "Baseline analysis of novel risk groups" is provided in Supplementary Results.
11. "To this end, the model was optimized to simultaneously score sleep stages, respiratory events, and desaturations while also achieving results comparable to previous polysomnographic foundation models14,15." Authors have only slightly compared their results for sleep staging with SOT studies. Authors could also compare their performance for respiratory and desaturation events detection with other studies.
12. Regarding these two sentences: "Importantly, these classification tasks are intermediate objectives designed to ensure that the model 'learns' a physiologically relevant feature space, forming the basis for our main goal of generating embeddings used towards risk group clustering." "indicating that the cluster assignments captured meaningful physiological differences not accounted for by simply classifying SDB severity using AHI". These two sentences are true, but apneic events, sleep stages, and oxygen desaturations are leading to these clusters. Thus, authors should indicate in the discussion that the risk groups found in their study are restricted to the physiological features related to the three classification tasks (sleep stages, oxygen desaturations, and apnea/hypopnea events).
13. The following two sentences are too vague: "These results indicate that RG4 patients have a continued elevated risk, particularly for cardiovascular diseases and neurologic outcomes." and "These findings suggest that RG3 patients have a moderately increased association of both cardiovascular and neurological conditions compared to RG2." Authors may consider improving or removing them from the manuscript.
14. If possible, diseases rows should appear in the same order both in Figure 3 and Supplementary Tables 1, 4, and 9.
15. Looking at Supplementary Table 6, why not the same comorbidities (all the comorbidities) are used as covariates for all the diseases?
16. Significance levels (*p<0.05; **p<0.01; ***p<0.001) should be included in the footnote of Table 1.
17. In model 5 and 6 from Table 1, RG4 has a higher CI of HR than RG4. "The consistent pattern of increasing HR point estimates underscores a strong association between higher-risk group classification and increased all-cause mortality, independent of potential confounders, including AHI". This warrants further discussion.
18. Authors should make an effort to better interpret differences between RG5 and RG3 and RG4.
19. What about validating (with and/or without fine-tuning) results on the SHHS and comparing them with SOTA studies? Another limitation is that the created Transformer-based model was not directly applied to SHHS, possibly due to a lack of generalizability.
20. The two-cluster approach, closely related to sleep fragmentation, suggests that sleep stage information plays the main role, although it is well known that apneic events and oxygen desaturations are related to it. This warrants further discussion.
21. Authors could include the following future goals: (i) to get informative clusters (risk groups) using subsets of reduced signals; (ii) to get informative clusters from models by directly targeting the diseases.

Version 1:

Reviewer comments:

Reviewer #1

(Remarks to the Author)
the authors have addressed all of our comments and we do not have any further revisions requested.

Reviewer #2

(Remarks to the Author)
I co-reviewed this manuscript with one of the reviewers who provided the listed reports. This is part of the Nature Communications initiative to facilitate training in peer review and to provide appropriate recognition for Early Career Researchers who co-review manuscripts.

Reviewer #4

(Remarks to the Author)
Thank you for the detailed responses. Two of the points previously raised still require clarification:
1. The authors state that source code will be shared "in accordance with IBM's internal regulations," but Nature

Communications requires that custom code central to the main claims be made available to editors and reviewers at submission, for example, through a private Code Ocean capsule. If internal regulations restrict public release, please explicitly state what can and cannot be shared publicly, and how reproducibility will be ensured.
2. The response to Comment #12 does not fully address the concern regarding the influence of supervised tasks. Because the embedding is learned through the supervised tasks of sleep staging, oxygen desaturation detection, and apnea and hypopnea classification, the representation is necessarily shaped by features predictive of these labels. The authors note that SSF is the strongest predictor of cluster membership, but it reflects sleep fragmentation and is itself a composite of EEG features related to arousals and stage transitions. This indicates that the embedding has learned a representation of EEG signals, but the underlying features remain driven by the training objectives.

Version 2:

Reviewer comments:

Reviewer #4

(Remarks to the Author)
The authors have addressed all of my previous comments, and I have no further concerns.

Open Access This Peer Review File is licensed under a Creative Commons Attribution 4.0 International License, which permits use, sharing, adaptation, distribution and reproduction in any medium or format, as long as you give appropriate credit to the original author(s) and the source, provide a link to the Creative Commons license, and indicate if changes were made.
In cases where reviewers are anonymous, credit should be given to 'Anonymous Referee' and the source.
The images or other third party material in this Peer Review File are included in the article's Creative Commons license, unless indicated otherwise in a credit line to the material. If material is not included in the article's Creative Commons license and your intended use is not permitted by statutory regulation or exceeds the permitted use, you will need to obtain permission directly from the copyright holder.
To view a copy of this license, visit https://creativecommons.org/licenses/by/4.0/

Re: Manuscript ID NCOMMS-25-23830-T,

"A Foundation Model for Sleep-Based Risk Stratification and Clinical Outcomes"

Point-by-point referee responses.

Referee 1

We thank Referee 1 for their detailed evaluation and constructive feedback. We note that the referee recognized the novelty of applying advanced AI to a large sleep cohort, stating that we "do a phenomenal job of leveraging advanced methods and cutting-edge technology … to identify unique clusters using physiologic parameters and predict long-term health outcomes." Several of the referee's major concerns overlap with the three key issues that guided our manuscript reorganization: novelty beyond AHI, external validation and robustness, and clinical and translational relevance.

1. **Novelty beyond AHI:** Referee 1 questioned whether our clusters were primarily driven by AHI or respiratory events, echoing their summary statement that the transformer model "appears to be predominantly driven by the apnea-hypopnea index (AHI)." As we clarify in our responses, we believe this was a misunderstanding driven by our placement of critical analyses in the Supplement. We now elevate these results into the main text and add new sensitivity analyses (EEG/ECG "zero-out" tests) that demonstrate multimodal contributions.
2. **External validation and robustness:** Referee 1 commented that "external validation is lacking" and raised concerns that clustering may reflect "noise or dataset-specific bias." We address this directly with the expanded SHHS validation, stability metrics, and propensity analyses, showing the reproducibility of our findings across cohorts and methods.
3. **Clinical and translational relevance:** Referee 1 suggested that while technically strong, the model "identifies patients with high AHI without substantially advancing our understanding of physiologic subtypes or impacting patient care." We address this concern by moving head-to-head AHI vs. embedding comparisons and disease-specific hazard ratios into the main text (Figures 1–3), we demonstrate that our clusters outperform AHI in predicting outcomes, establishing clear clinical relevance. Additionally, we add Figure 4 showing the complex patient mapping between AHI classifications and the identified risk groups, further demonstrating that the risk groups are not a recapitulation of AHI categories.

With this context, we provide detailed point-by-point responses to all comments from Referee 1, highlighting how the revised manuscript addresses both major and minor concerns.

1. "While the OSA diagnostic criteria incorporate AHI cutoffs there are other factors. It is not completely accurate to state that the criterion is solely based on AHI. Please adjust the language to match and reference the AASM ICSD-3-TR."

   Response: We agree with the referee that the previous statement about the role of AHI lacked proper nuance. We changed the wording in the Introduction, first paragraph to clarify this point and added the reference the referee suggested. Specifically, the text now reads, "Yet, assessment of sleep

integrity remains limited because clinical interpretation of polysomnography (PSG), the gold-standard diagnostic test, is often reduced to the apnea-hypopnea index (AHI)[3]. While AHI is central to current diagnostic criteria for sleep-disordered breathing as defined by the American Academy of Sleep Medicine (AASM ICSD-3-TR), it captures only a narrow slice of sleep physiology and carries limited prognostic value."

2. "Please include the number of samples used for training and testing in the results."

   Response: This information is included in the manuscript in the Model training narrative, specifically: "We trained the PSG foundation model on 9,203 studies using a multi-task objective designed to capture physiologic features across multiple domains. The foundation model was designed to address three tasks: sleep stage classification, respiratory event detection (e.g., apneas and hypopneas), and oxygen desaturation identification. Of the available PSGs, 30 were reserved for validation and 500 for testing with inclusion criteria detailed in Supplementary Methods: Data Quality Assessment."

3. "How many PSGs passed the quality assessment? Were there differences in the population of patients whose PSGs were included in the analysis versus were excluded? This exclusion for seemingly random "quality" issues could systematically bias the studied sample."

   Response: We have added the following statement under Data quality assessment: "A total of 267 files that were not artifact-free in all three sleep stages were excluded. No major demographic differences were observed between the excluded samples and the ones that were kept."

4. "Do you feel the average TST of under 100 minutes in the RG5 group is problematic? As this group represents the highest risk group upon which much of the conclusions/discussion is centered, we should be confident about the data quality in this subgroup. AASM standards for diagnostic PSG (minimum number of channels for at least 2 hours) should be part of the quality assessment and inclusion in the study. While it may be significant that these participants have short in-lab sleep times, inclusion of PSG with such limited data does not help answer the premise of the study which is to identify physiologic measures embedded within a PSG that can cluster patients into different risk groups."

   Response: The average total sleep time of 98±89 minutes in RG5 is lower than the other risk groups which likely reflects inherent and unique sleep pathophysiology in part likely attributable to greater sleep apnea burden. While the AASM standards do recommend a minimum of 2 hours for a diagnostic sleep study, we believe that multi-channel quality data for approximately 100 minutes is sufficient in scope, particularly given the approach taken to generate high dimensional representations.

5. "Include baseline characteristics for the overall study population (either in a separate table or as a column in supplementary Table 7)."

   Response: Added Supplementary Table 6 demographics for the study population.

6. "In supplemental Table 7 please include a row for OSA diagnosis."

   Response: We have added the requested rows to Supplementary Table 7.

7. "How was PAP therapy defined; seems it was based on EHR scripts for PAP, is this correct? Did this include bilevel, ASV, NIV etc.?"

   Response: PAP therapy was defined as the presence of an order for a positive airway pressure device in the electronic health record. For each patient, the initiation date of the PAP order was recorded when available. This definition encompasses devices with PAP configuration, including bilevel, ASV

and NIV. Thus, our analysis was based on whether PAP therapy was prescribed, rather than on adherence or actual device usage. We extended this clarification in the text.

8. "The statement that PAP therapy did not alter the results should be softened. If PAP was identified by EHR prescriptions, actual PAP use (or lack of use) cannot be assessed and "PAP treatment" may represent other clinical characteristics entirely. As such, baseline characteristics for the PAP and non-PAP groups should be presented. Was PAP mostly a surrogate for moderate-to-severe OSA diagnosis or OSA + CVD/AFIB/HTN etc.?"

   Response: We agree and have clarified throughout the manuscript that PAP exposure is inferred only from EHR prescription data, and thus we cannot determine adherence. This limitation is now explicitly stated in both the Methods and Discussion sections. While PAP prescriptions likely act as a proxy for more severe OSA and cardiometabolic comorbidities, our analyses show that excluding PAP-prescribed patients (Table 1, Model 5) did not materially alter the hazard ratios across risk groups. Similarly, restricting analyses to patients ≥55 years old (Table 1, Model 6) produced consistent results, underscoring that the observed prognostic signal is robust across clinically relevant subgroups. These findings demonstrate that our results are not explained by PAP exposure alone. This point is important, because it directly counters the concern that the lack of predictive value of AHI categories could be attributed to PAP treatment effects.

9. "Why was validation only provided for the classifier by sleep fragmentation? How is this classifier related to the 5-cluster and 2-cluster embedding-based classifiers? Please provide external validation for the embedding-based foundation model classifier. Without validation of the transformer-based cluster method, there is decreased enthusiasm for the validity of the results."

   Response: In addition to the validation provided through the sleep fragmentation proxy classifier we have also performed a validation of the entire pipeline by applying our foundation model directly to the SHHS raw PSG data. The results of this validation are described under section External validation.

10. "The lack of precision in PAP metrics may bias toward the null as stated. However, the overall discussion of "PAP use" in the study is confounded by the risk profile inherent in a clinical cohort of patients that are prescribed PAP. In model 5 when patients with PAP therapy prescription were excluded, HR was attenuated in RG5 v RG1. This could suggest that PAP therapy increases risk for mortality in the highest risk group (or decreases risk in low risk RG1). Alternatively, PAP prescriptions may just be a surrogate for worse health overall. I would leave the statement that lack of precise PAP data is a limitation, and not comment on which direction it would bias the results. This also raises another interesting point which is that you report no effect of AHI on outcomes, but this is only in a population that may have been treated with CPAP. You could exclude PAP users (as done in model 5) and retest the association between AHI and outcomes (though given these were clinical PAP prescription, essentially all moderate-to-severe AHI may have been prescribed PAP)."

    Response: We thank the reviewer for raising the important issue of PAP therapy as a potential confounder. As noted, PAP prescription tends to select higher-risk individuals, and incomplete adherence data can obscure its true effect. We agree it is more appropriate not to assign directionality to this bias without further evidence.

    Regarding the attenuation of hazard ratios when excluding PAP-treated patients, this can be explained by differential removal of healthier, PAP-treated individuals disproportionately from lower-risk groups (e.g., RG1). As suggested, we re-analyzed the association between AHI and outcomes after excluding PAP-treated patients and found no meaningful change in our conclusions. This limitation is acknowledged, as some patients may have obtained therapy outside our system.

Importantly, the independent SHHS validation, a prospective study with treatment carefully controlled, confirms that risk-group stratification provides a stronger predictor of survival than AHI. These findings suggest that PAP confounding does not account for the observed superiority of our risk groups.

We have added the following texts to the manuscript: "By contrast, AHI-defined categories of mild, moderate, and severe sleep apnea showed no association with mortality, underscoring the added value of the embedding-derived risk groups. A Cox model substituting AHI categories for risk groups (with the same covariates) showed no increase in mortality relative to AHI < 5 (Figure 3), highlighting that traditional severity thresholds do not explain the observed survival differences. For example, in the case of all-cause mortality, there was no significant increase in risk compared to the baseline group, even after excluding patients with available CPAP treatment information: HR=0.79 (95% CI 0.60–1.03), HR=0.70 (95% CI 0.49–1.01), and HR=1.05 (95% CI 0.71–1.55) for mild, moderate, and severe AHI, respectively."

Commented [EB1]: Added in-line the results for mortality without the subjects with PAP therapy as requested by reviwers

Commented [JR2R1]: Thank you for the clarification.

11. "Describe the NLP process used to abstract clinical data, including validation and/or accuracy. Which measures specifically were extracted from EHR using NLP? Were the study outcomes or MACE determined by NLP? What was the adjudication process for these outcomes/events? If not done, a random sample should be selected for testing the NLP process."

    Response: To clarify, our study did not rely on traditional natural language processing (NLP) for extracting polysomnographic data. Instead, we extract the PSG metrics directly from the structured HTML format of the final polysomnography reports. Each study report is based on a standardized HTML template from Polysmith Software in which all metrics (e.g., AHI Total, AHI Off-supine, etc., AHI Supine, Arousal Index, CAI, central apneas, hypopneas, maximum $EtCO_2$, mean and minimum O_2 saturation, NREM/REM-specific indices, sleep stage percentages, snoring, and sleep times) are recorded within predefined identified <div> tags. This formatting enabled deterministic HTML parsing, rather than relying on NLP techniques. Moreover, this dataset is routinely curated by humans who verify that the extracted metrics match the reported values. The clinical outcomes, including major adverse cardiovascular events (MACE), were not determined using parsing or NLP. These outcomes were based on a predefined set of ICD codes in the electronic health record. These points were clarified and added to the text.

12. "How does the foundational model account for poor quality signals in channels other than EEG (described in the supplement)?"

    Response: The following conditions need to be satisfied at run time for each 30 second epoch in addition of the global criteria for the EEG channels:

- Median absolute deviation of the EEG or EKG is bigger than zero
- EEG channels don't contain absolute values bigger than 10 after normalization
- SPO2 channel doesn't contain values outside of its normal range

13. "What was the percentage of missing data that required imputation? What imputation strategy was used and were assumptions tested for the selected strategy?"

    Response: To address this we included the following section in the manuscript: "Missing Data and Imputation". Missing data were addressed using imputation strategies tailored to the extent of missingness. Variables with <5% missingness (*e.g.*, BMI, smoking status) were imputed by mean or mode substitution, while variables with >5% missingness (*e.g.*, comorbidity indicators) were multiply

imputed using chained equations. Imputation models incorporated age, sex, BMI, and comorbidities to preserve relevant associations.
Robustness was confirmed by comparing hazard ratios from complete-case analyses with those obtained from imputed datasets, which showed consistent results in both magnitude and direction. This approach ensured that missing data did not introduce systematic bias into the associations between risk groups and clinical outcomes."

14. "Rather than describing your methods, results, and conclusions in the final paragraphs of the introduction, would recommend stating your hypotheses and leaving these other pieces (except for some general methods) for the appropriate sections."

    Response: Thank you for the suggestion. The entire text has been reorganized and rewritten to improve the presentation and to incorporate clarifying results.

15. "First two paragraphs of the results seem like introduction and methods."

    Response: The Results section has been reorganized, with several new figures, and the initial paragraph has been replaced.

16. "Another limitation is relying on EHR based ICD-10 diagnostic codes. This should be included in the limitations paragraph."

    Response: We added this limitation in the discussion.

17. "First, the transformer model is trained on three tasks that are primarily respiratory signal–based, ignoring the complex interplay between other physiologic channels, such as cardiac and limb movement data. As a result, the derived clusters appear to be predominantly driven by the apnea-hypopnea index (AHI), offering limited novelty beyond known severity-based classifications and potentially steering the field in the direction of prior epidemiologic evidence which has not been supported in randomized trials."

    Response: We agree that this is a key point, and we have reorganized the manuscript to better highlight our findings that the reported clusters are conveying different information than the AHI categories. We find the AHI categories do not provide any meaningful correlations with outcomes (Figure 3), while the risk groups we report are predictive of outcomes (Figure 1). We also show in Figure 4 that the patient level mapping between individuals in the AHI categories and the risk groups is a complex, nonlinear relation.

18. "All long-term outcomes are grouped together in the analysis, with endpoints trending uniformly in one direction across clusters. There is no attempt to differentiate clusters based on specific outcome patterns—e.g., some being more associated with neurological outcomes and others with cardiac events—missing an opportunity for a more nuanced understanding."

    Response: The ordinal ranking of the clusters is an important feature that is discussed along with the risk groups and comparisons between them in the reorganized and rewritten Results-Novel Sleep Profiles section. There are three subsections covering and comparing the health risks and characteristics of each embedding-derived cluster. Specifically, the subsections are now Cluster Embedding into Risk Groups, Associations between Risk Groups and Incidence of Clinical Outcomes, and All-Cause Mortality. We have also added the Sankey diagram (new Figure 4) mapping between AHI categories and risk groups (R1 to R5) to show the complicated relationship between these risk groups and the AHI categories.

19. “The validation strategy is also limited. The external validation cohort only confirmed the two-cluster solution, not the five-cluster model that is emphasized in the main findings, which reduces the rigor and generalizability of the five-cluster interpretation.”

    Response: We performed the five-cluster analysis on the external data set, SHHS, finding that the results hold. This analysis is described in the updated text.

20. “Furthermore, within the validation cohort, only heart failure and all-cause mortality were validated as outcomes, omitting other clinically relevant endpoints. This weakens the argument that the clustering is broadly predictive or clinically actionable.”

    Response: This concern about the validation and generalizability identified by both referees. The results we reported cover those published in a specific analysis of SHHS that studied heart-failure and mortality. Our intention is to validate our results on an external data set, not to perform a comprehensive study covering a body of research. Certainly, that is interesting and would be a contribution to the community, but it is beyond the scope of the work we are reporting.

21. “The clustering approach also fails to incorporate important clinical and behavioral variables such as medications, sleep duration, sleep timing, and lifestyle factors, all of which play a major role in sleep health and are routinely overlooked in practice. Their exclusion further limits the translational value of the clusters.”

    Response: We agree that medications, sleep duration/timing, and lifestyle factors are important determinants of sleep health and clinical outcomes. Our clustering framework, however, was intentionally designed as a data-driven physiologic approach, deriving embeddings directly from raw PSG signals rather than relying on predefined clinical or behavioral covariates. This strategy avoids bias introduced by assumptions about which factors should be most relevant, instead allowing the data to reveal physiologic subtypes. The validity of this approach is reflected in its performance: embedding-derived clusters robustly stratify mortality and disease incidence, whereas AHI categories do not. We view this as complementary to models that incorporate medications and lifestyle factors, and agree that future work could integrate such variables to extend the translational scope. Indeed, we highlight this in the Discussion as an important future direction.

22. “Ultimately, while the technical implementation is strong, the resulting model identifies patients with high AHI without substantially advancing our understanding of physiologic subtypes or impacting patient care. The clustering does not integrate symptom burden or treatment responsiveness, which are central to current precision sleep medicine.”

    Response: We thank the referee for recognizing the innovation in our technical approach and call their attention to Figures 1 and 3 that directly compare the disease outcome predictions between the embedding-derived risk groups we report and AHI categories. These figures demonstrate that the risk groups have significant correlations with specific outcomes, while the AHI categories are not predictive. In this way the risk groups have direct clinical relevance. Additionally, Figure 4 shows that the connection between patients is the AHI categories and the risk groups is a complicated mapping.

23. “Despite the use of advanced AI tools, the clinical and translational contribution is modest.”

    Response: We have added Figures 1-3 that demonstrate that the identified embedding clusters act as risk groups that are predictive of clinical outcomes missed by the existing AHI categories. As a result, there is a significant direct clinical contribution.

24. "AHI remains a useful clinical measure, so the comparison must be explicit."
Response: Addressed with new hazard ratio analyses for AHI (Figure 3) directly compared to embedding-derived groups (Figure 1).

25. "The AI methods appear to be a repackaging of existing models."
Response: Addressed by highlighting the novel tokenization strategy for long PSG time-series (Results, Figure 5; Discussion).

26. "The clinical impact of the clusters is not clear."
Response: Addressed by presenting hazard ratios (Figure 1) and survival analyses (Figure 2), which demonstrate strong, clinically interpretable differences in risk.

Referee 2

"I co-reviewed this manuscript with one of the reviewers who provided the listed reports. This is part of the Nature Communications initiative to facilitate training in peer review and to provide appropriate recognition for Early Career Researchers who co-review manuscripts."

Referee 3

We thank Referee #2 for their thorough and constructive evaluation. Their review focused on three main areas: (1) clarification of methodological details in model training, clustering, and reproducibility; (2) further benchmarking and interpretation of our model's performance, including the physiologic basis of the clusters; and (3) requests for expanded discussion of limitations and future directions.

Many of these points are requests for clarification (*e.g.*, training sample sizes, artifact handling, hardware/software used, covariate selection, and figure/table consistency), which we have now incorporated into the revised manuscript. Importantly, several of Referee #2's comments relate to the broader concern about novelty, that our model might be a repurposed RoBERTa architecture, or that clusters are largely recapitulating apnea/hypopnea events, desaturations, or staging. We address this directly by elevating analyses from the Supplement into the main text, highlighting the novel tokenization strategy for multimodal PSG data and new results showing the importance of non-respiratory signals (*e.g.*, EEG, ECG) and Spectral Sleep Fragmentation (SSF) as leading features.

Other concerns connect to robustness and generalizability. Referee #2 noted the original validation was limited to a two-cluster summary; we have now extended validation of the five-cluster framework in the external SHHS cohort and confirmed cluster stability across metrics. Finally, we acknowledge the referee's emphasis on translational value. We have strengthened the manuscript by presenting disease-specific hazard ratios (Figure 1) and survival curves (Figure 2) in the main text, and by explicitly comparing embedding-derived groups to AHI categories; showing that AHI fails to predict outcomes where our risk groups succeed.

Together, these changes provide clarity on methodological novelty, confirm external robustness, and demonstrate clinical relevance, while also addressing each of the referee's detailed points below.

1. "There are some statements without a single reference. For example, only one reference is provided within the first two paragraphs of the introduction."

   Response: Thank you. References have been added, including in the Introduction, and reorganized throughout the manuscript.

2. “Figure 1 should graphically include all the PSG channels used.”
   Response: Figure 1 (now Figure 6) depicts all the channel categories used and then lists key channel in the caption. We ensured all channels (e.g., C4, F4, SpO2) are explicitly noted.

3. “Why do authors choose oxygen desaturations and not arousals or spindles detection? Further explanations should be provided.”
   Response: We appreciate the reviewer’s question. Our analyses relied on all human-annotated data available from the sleep laboratory, which included apneas, hypopneas, sleep stages, and oxygen desaturations. While arousals and spindles are used in specific scoring tasks, such as identifying transitions between sleep stages and characterizing NREM sleep architecture, they are not routinely recorded in a structured format and were therefore not available for inclusion in our analysis.

4. “Authors perform a multi-task (sleep stages, oxygen desaturations, and respiratory events) model. Why do author shave choose it over single-task models (i.e., separate models and clusters for each task)? Further explanations should be provided to better highlight the advantages of multi-task learning. I leave as optional/future task to perform separate clustering approaches for three single-task models. This could help to support the suitability of multi-task learning for finding better clusters.”
   Response: Sleep stages, respiratory events, and desaturations are physiologically interrelated, and shared representations are better suited to capture these complex relationships than separate models. In addition, a multitask approach is computationally more efficient, which is an important consideration given the scale of our dataset and the available computational resources. While we agree that comparing single-task clustering could provide valuable insights, it remains an area for future work as we had to prioritize feasibility and efficiency in this initial study.

5. “Why do authors only use artifact-free PSGs? DL methods are able to deal with artifacts.”
   Response: While deep learning models can tolerate some level of artifacts, severe artifacts can degrade performance and obscure meaningful physiologic patterns. Therefore, we applied rigorous quality control procedures to ensure reliable training signals. Our artifact detection pipeline allowed up to 40% artifacts per channel, which balanced the need to retain most of the data while maintaining signal integrity. High-quality data is especially critical for training large models, such as LLMs, where noise can accumulate and impair representation learning. This principle is well established in foundation model development (ref: https://arxiv.org/abs/2107.06499)

6. “With respect to model training: (i) why do authors stop it at epoch 50 rather than using early stopping? (i) why do authors use so few validation and test subjects? Further explanations should be provided.”

   Response: We trained the model for a fixed 50 epochs and selected the best-performing checkpoint based on validation performance, rather than using early stopping. This approach avoids the instability that can arise from early stopping in noisy physiological time series data and ensures a consistent training procedure. While the validation set includes 30 subjects and the test set includes 500, each subject contributes hundreds of 30-second segments, resulting in tens of thousands of samples overall. This provides sufficient statistical power for both model selection and evaluation. We also emphasize the distinction between the two sets: the validation set is used during training to select the optimal checkpoint, while the test set is held out entirely for final performance evaluation, ensuring an unbiased assessment of generalization.

7. “I would include a figure to fully explain the clustering approach in a graphical way. This figure could be included in the main manuscript or as a supplementary material/file, and it should describe every step with input-output dimensions.”

   Response: We appreciate the reviewer’s suggestion to include a schematic of the clustering method. However, the procedure is essentially a simple projection of study-level embeddings to a fixed-length

representation followed by standard k-means clustering. We feel that a figure would not provide additional clarity beyond the description already included in the Methods. To avoid overcomplicating the presentation, we prefer to keep the explanation in text form. The following description was added to the manuscript for clarity: "To enable clustering of variable-length PSG embeddings, we transformed each study into a fixed-size feature vector suitable for k-means analysis. After model training, the CLS token for each 30-second epoch was stacked to create a two-dimensional embedding matrix with 768 rows and a variable number of columns reflecting PSG duration. Because study length differed across patients, k-means clustering could not be applied directly. To address this, we calculated pairwise distances between each embedding and all other embeddings by aligning rows (time dimension) and averaging across the feature dimension. This process produced a fixed length distance vector for each study, which was then used as input for clustering."

8. "What is the hardware equipment used to train the models? This can help readers know the resources need to reproduce the proposed methodology."

   Response: We have trained the model on two NVIDIA A40 GPUs each with 46Gb of memory. The following text was added to the manuscript: "The model was implemented in PyTorch using the HuggingFace model library, and training was performed on two NVIDIA A40 GPUs with 46 GB memory each.""

9. "What is the software used to train the Transformer model? At least, authors should include the code of the foundation model training and embedding generation, as well as the clustering method. In this way, authors could reproduce the proposed methodology using other datasets such as SHHS."

   Response: The model was implemented in Pytorch using HuggingFace model library. The following text was added to the manuscript: "The model was implemented in PyTorch using the HuggingFace model library, and training was performed on two NVIDIA A40 GPUs with 46 GB memory each." In addition, we commit to sharing code relevant to model training, embedding generation, and clustering in accordance with IBM's internal regulations and copyright policies, ensuring that reproducibility is balanced with compliance to institutional requirements.

10. "At the end of 'Cluster Embeddings into Risk Groups' authors should indicate that a 'Baseline analysis of novel risk groups' is provided in Supplementary Results."

    Response: The following text was added to the manuscript under the suggested section: "Results from Cox regression and propensity score matching, described below, further supported the stability and clinical relevance of these assignments. Baseline analysis of novel risk groups is provided in Supplementary Results."

11. "'To this end, the model was optimized to simultaneously score sleep stages, respiratory events, and desaturations while also achieving results comparable to previous polysomnographic foundation models14,15.' Authors have only slightly compared their results for sleep staging with SOT studies. Authors could also compare their performance for respiratory and desaturation events detection with other studies."

    Response: We appreciate the referee's suggestion. Our study, however, is not intended as a benchmarking exercise against prior sleep-staging or respiratory-event detection models. Instead, the central novelty lies in moving beyond event detection to discover physiologic subtypes with predictive value for long-term outcomes. To avoid overstating, we have clarified in the revised manuscript that while our foundation model can perform these conventional detection tasks, our contribution is in deriving embedding-based risk groups that stratify mortality and incident disease more effectively than AHI**.**

That said, we have expanded the Methods and Discussion to explicitly note that our model achieves performance on par with prior work for sleep staging and respiratory event detection (see refs. 14,15), thereby establishing a baseline. However, rather than replicate detailed benchmarking for desaturations and respiratory events, which are well covered in the cited literature, we emphasize the new results that these embeddings carry prognostic information beyond traditional PSG metrics.

12. "Regarding these two sentences: 'Importantly, these classification tasks are intermediate objectives designed to ensure that the model 'learns' a physiologically relevant feature space, forming the basis for our main goal of generating embeddings used towards risk group clustering.' 'indicating that the cluster assignments captured meaningful physiological differences not accounted for by simply classifying SDB severity using AHI'. These two sentences are true, but apneic events, sleep stages, and oxygen desaturations are leading to these clusters. Thus, authors should indicate in the discussion that the risk groups found in their study are restricted to the physiological features related to the three classification tasks (sleep stages, oxygen desaturations, and apnea/hypopnea events)."

    Response: We respectfully disagree that the risk groups are restricted to these three inputs. As described in the subsection *Predicting Clusters Using Standard PSG Metrics* and shown in Supplementary Table 4, when we attempted to approximate embedding-derived clusters with conventional PSG variables, the leading predictor across all cluster solutions was *Spectral Sleep Fragmentation (SSF)*. SSF is a novel measure quantifying rapid stage transitions in the EEG spectrum and is not simply an apnea/hypopnea, desaturation, or staging variable. Its consistent prominence demonstrates that the model captures physiologic patterns beyond the three classification tasks noted by the referee. This directly supports our central point: the embeddings identify physiologic dimensions not well characterized by conventional metrics, which is why the risk groups stratify outcomes where AHI categories fail.

13. "The following two sentences are too vague: 'These results indicate that RG4 patients have a continued elevated risk, particularly for cardiovascular diseases and neurologic outcomes.' and 'These findings suggest that RG3 patients have a moderately increased association of both cardiovascular and neurological conditions compared to RG2.' Authors may consider improving or removing them from the manuscript."

    Response: The statements highlighted by the referee have been rewritten as part of reorganizing the text in the Results section, to clarify interpretation of RG3 and RG4 with specific outcomes and hazard ratios reported.

14. "If possible, diseases rows should appear in the same order both in Figure 3 and Supplementary Tables 1, 4, and 9."

    Response: We reorganized these tables for consistency. In the submitted version, Figure 3 had diseases alphabetized within disease categories. All others have been rectified for consistency: Supplementary Table 4 is now Figure 3; Supplementary Table 1; Supplementary Table 9 is now Supplementary Table 8.

15. "Looking at Supplementary Table 6, why not the same comorbidities (all the comorbidities) are used as covariates for all the diseases?"

    Response: We selected the covariates per comorbidity based on the biological plausibility of the comorbidities that often occur with the disease in question which could be potentially confounding.

16. "Significance levels (*p<0.05; **p<0.01; ***p<0.001) should be included in the footnote of Table 1."

    Response: Thank you for highlighting this oversight. We have added the appropriate significance level description into the Table 1 legend now.

17. “In model 5 and 6 from Table 1, RG4 has a higher CI of HR than RG4. ‘The consistent pattern of increasing HR point estimates underscores a strong association between higher-risk group classification and increased all-cause mortality, independent of potential confounders, including AHI’. This warrants further discussion.”

    Response: We thank the referee for pointing out the variability in hazard ratio confidence intervals in Table 1, Models 5 and 6. We have revised the Results text to avoid overstating the monotonic pattern and now describe the findings as a general ordinal trend in risk across RG2–RG5, with RG5 consistently at the highest risk. This revision ensures that the interpretation accurately reflects the statistical results while still underscoring the robust stratification captured by the embedding-derived groups.

18. “Authors should make an effort to better interpret differences between RG5 and RG3 and RG4.”
    Response: In section Results-Novel Sleep Profiles we have reorganized and rewritten the text into three subsections covering and comparing the health risks and characteristics of each embedding-derived cluster. Specifically, the subsections are now Cluster Embedding into Risk Groups, Associations between Risk Groups and Incidence of Clinical Outcomes, and All-Cause Mortality. We have also added the Sankey diagram mapping between AHI categories and risk groups (R1 to R5) to show the complicated relationship between these risk groups and the AHI categories.

19. “What about validating (with and/or without fine-tuning) results on the SHHS and comparing them with SOTA studies? Another limitation is that the created Transformer-based model was not directly applied to SHHS, possibly due to a lack of generalizability.”

    Response: This comment details aspects of one the major concerns emerging from the review process, external validation and generalizability. We have now applied the full pipeline we developed to the external SHHS dataset, and validate our results. Survivability curves are presented in Figure 2

20. “The two-cluster approach, closely related to sleep fragmentation, suggests that sleep stage information plays the main role, although it is well known that apneic events and oxygen desaturations are related to it. This warrants further discussion.”

    Response: We clarified the two-cluster results and now define Spectral Sleep Fragmentation (SSF*)*, which emerged as the leading feature across cluster solutions (Supplementary Table 4). The new section Predicting Clusters Using Standard PSG Metrics shows that SSF can approximate a two-cluster split, but conventional PSG measures cannot reproduce the richer stratification of the five-cluster embedding framework, underscoring that our findings extend beyond apnea and desaturation events.

21. “Authors could include the following future goals: (i) to get informative clusters (risk groups) using subsets of reduced signals; (ii) to get informative clusters from models by directly targeting the diseases.”

    Response: We agree with the referee that future work could explore reduced-signal models and disease-targeted clustering. In part, we already address (i) in the current manuscript through the Predicting Clusters Using Standard PSG Metrics section, where we approximate the embedding-derived risk groups using conventional features. Spectral Sleep Fragmentation (SSF), in particular, emerged as the leading feature across cluster solutions (Supplementary Table 4). However, performance drops substantially beyond the two-cluster split, underscoring that reduced-signal approaches cannot fully capture the richer physiologic structure learned by the embeddings. As we note in the Discussion, future work may incorporate both reduced-signal models and disease-specific objectives, but our present results establish the value of an unbiased, data-driven approach that generalizes across multiple disease outcomes.

Point-by-Point Response to Reviewers' Comments

Nature Communications manuscript NCOMMS-25-23830A-Z

Title: A Foundation Model for Sleep-Based Risk Stratification and Clinical Outcomes

We thank the Editor and Reviewers for their careful evaluation of our manuscript and for the opportunity to revise our work. Below we provide our detailed responses and a description of the specific changes made in the revised manuscript. All changes have been highlighted in the revised manuscript using track changes.

A. Reviewer #1

Comment: *"The authors have addressed all of our comments and we do not have any further revisions requested."*

Response: We thank Reviewer #1 for their thorough review and for confirming that all prior comments have been satisfactorily addressed. No further action was required.

B. Reviewer #2

Comment: *"I co-reviewed this manuscript with one of the reviewers who provided the listed reports. This is part of the Nature Communications initiative to facilitate training in peer review and to provide appropriate recognition for Early Career Researchers who co-review manuscripts."*

Response: We thank Reviewer #2 for their contribution to the review process. No specific revisions were requested.

C. Reviewer #4

We thank Reviewer #4 for their detailed follow-up and for clarifying the remaining points requiring attention. Each issue is addressed below.

Reviewer #4, Comment 1

Comment: *"The authors state that source code will be shared 'in accordance with IBM's internal regulations,' but Nature Communications requires that custom code central to the main claims be made available to editors and reviewers at submission, for example, through a private Code Ocean capsule. If internal regulations restrict public release, please explicitly state what can and cannot be shared publicly, and how reproducibility will be ensured."*

Response: We appreciate this clarification and have fully addressed this concern.

What has changed:

1. Reviewer access to code:
   All custom code central to the manuscript's main claims has now been deposited in a

private Zenodo record, which allows anonymous, reviewer-only access via a secure link.

2. Scope of code availability clarified:
   The Zenodo archive includes:

   a) A detailed README with installation instructions and expected outputs
   b) Model architecture implementation
   c) Training and inference scripts
   d) Clustering and outcome-analysis code
   e) Representative example data

   This enables editors and reviewers to fully assess methodological validity and reproducibility.

3. Public vs restricted components explicitly stated:
   We now clearly state that:

   a) Code is available to reviewers and can be shared publicly subject to institutional approval from IBM and Cleveland Clinic.
   b) Raw clinical data from Cleveland Clinic cannot be publicly released due to IRB and Data Use Agreement restrictions.
   c) Derived data and summary-level results may be shared upon reasonable request, subject to institutional approval.

4. Reproducibility pathway:
   Reproducibility is ensured through:

   a) Full access to model code and pipelines
   b) Use of publicly available SHHS data for external validation
   c) Detailed methodological descriptions in the manuscript and Supplementary Methods

Manuscript changes:

- Updated Code Availability section
- Updated Data Availability section
- Corresponding updates in the Software Policy and Machine Learning Checklist

Reviewer #4, Comment 2

Comment: *"The response to Comment #12 does not fully address the concern regarding the influence of supervised tasks. Because the embedding is learned through the supervised tasks of sleep staging, oxygen desaturation detection, and apnea and hypopnea classification, the*

representation is necessarily shaped by features predictive of these labels. The authors note that SSF is the strongest predictor of cluster membership, but it reflects sleep fragmentation and is itself a composite of EEG features related to arousals and stage transitions. This indicates that the embedding has learned a representation of EEG signals, but the underlying features remain driven by the training objectives."

Response: We agree with the reviewer's characterization and have revised the manuscript to explicitly acknowledge and clarify this point.

Key clarifications added:

1. Explicit acknowledgment of supervised influence: We now clearly state that the learned embedding is not label-independent and is necessarily shaped by the supervised objectives (sleep staging, respiratory events, desaturation detection).
2. Clarification of intent: The supervised tasks are framed explicitly as physiological constraints, not endpoints. Their role is to:

    a) Anchor representation learning in clinically validated PSG phenomena
    b) Reduce dependence on any single annotation system
    c) Encourage learning of shared, multimodal physiological structure

3. Why embeddings go beyond task labels: We provide multiple lines of evidence showing that:

    a) Conventional PSG features derived from the same annotations cannot reproduce the cluster structure
    b) Multimodal ablation (EEG and ECG zeroing) significantly disrupts cluster assignment
    c) External validation in SHHS reproduces outcome stratification despite lower-resolution PSG and different scoring protocols

4. SSF interpretation refined: SSF is now explicitly described as:

    a) A downstream, interpretable correlate of embedding structure
    b) Not a direct optimization target
    c) Insufficient on its own to reconstruct higher-order clustering beyond simple separations

5. Future directions stated: We now explicitly note that future work will explore:

    a) Self-supervised and disease-targeted objectives
    b) Reduced reliance on technician-defined labels

Manuscript changes:

- Revised Discussion section (limitations and interpretation)
- Expanded clarification in Methods (Model Training and Embedding Generation)
- Additional language in Sensitivity Analyses and Discussion

Manuscript #: NCOMMS-25-23830-T

Title: A Foundation Model for Sleep-Based Risk Stratification and Clinical Outcomes

Summary:

This study identifies clusters of patients in a clinical cohort that underwent PSG testing. These clusters are generated by applying unsupervised machine learning (K-means) to embeddings extracted from PSG by a transformer-based model. Survival analysis/time-to-event analysis was performed to assess the risk of poor health outcomes among specific clusters as compared to the reference cluster. The authors should be congratulated on applying this novel technique is a large clinical sleep cohort. However, there are several limitations which must be addressed.

Major:

- Intro:
  - o While the OSA diagnostic criteria incorporate AHI cutoffs there are other factors. It is not completely accurate to state that the criterion is solely based on AHI. Please adjust the language to match and reference the AASM ICSD-3-TR.
- Results:
  - o Please include the number of samples used for training and testing in the results.
  - o How many PSGs passed the quality assessment? Were there differences in the population of patients whose PSGs were included in the analysis versus were excluded? This exclusion for seemingly random “quality” issues could systematically bias the studied sample.
  - o Do you feel the average TST of under 100 minutes in the RG5 group is problematic? As this group represents the highest risk group upon which much of the conclusions/discussion is centered, we should be confident about the data quality in this subgroup. AASM standards for diagnostic PSG (minimum number of channels for at least 2 hours) should be part of the quality assessment and inclusion in the study. While it may be significant that these participants have short in-lab sleep times, inclusion of PSG with such limited data does not help answer the premise of the study which is to identify physiologic measures embedded within a PSG that can cluster patients into different risk groups.
  - o Include baseline characteristics for the overall study population (either in a separate table or as a column in supplementary Table 7).
  - o In supplemental Table 7 please include a row for OSA diagnosis
  - o How was PAP therapy defined; seems it was based on EHR scripts for PAP, is this correct? Did this include bilevel, ASV, NIV etc.?
  - o The statement that PAP therapy did not alter the results should be softened. If PAP was identified by EHR prescriptions, actual PAP use (or lack of use) cannot be assessed and “PAP treatment” may represent other clinical characteristics entirely. As such, baseline characteristics for the PAP and non-PAP groups

should be presented. Was PAP mostly a surrogate for moderate-to-severe OSA diagnosis or OSA + CVD/AFIB/HTN etc.?

  - **Why was validation only provided for the classifier by sleep fragmentation? How is this classifier related to the 5-cluster and 2-cluster embedding-based classifiers? Please provide external validation for the embedding-based foundation model classifier. Without validation of the transformer-based cluster method, there is decreased enthusiasm for the validity of the results.**

- Discussion
  - The lack of precision in PAP metrics may bias toward the null as stated. However, the overall discussion of "PAP use" in the study is confounded by the risk profile inherent in a clinical cohort of patients that are prescribed PAP. In model 5 when patients with PAP therapy prescription were excluded, HR was attenuated in RG5 v RG1. This could suggest that PAP therapy increases risk for mortality in the highest risk group (or decreases risk in low risk RG1). Alternatively, PAP prescriptions may just be a surrogate for worse health overall. I would leave the statement that lack of precise PAP data is a limitation, and not comment on which direction it would bias the results. This also raises another interesting point which is that you report no effect of AHI on outcomes, but this is only in a population that may have been treated with CPAP. You could exclude PAP users (as done in model 5) and retest the association between AHI and outcomes (though given these were clinical PAP prescription, essentially all moderate-to-severe AHI may have been prescribed PAP).
- Methods
  - Describe the NLP process used to abstract clinical data, including validation and/or accuracy. Which measures specifically were extracted from EHR using NLP? Were the study outcomes or MACE determined by NLP? What was the adjudication process for these outcomes/events? If not done, a random sample should be selected for testing the NLP process.
  - How does the foundational model account for poor quality signals in channels other than EEG (described in the supplement)?
  - What was the percentage of missing data that required imputation? What imputation strategy was used and were assumptions tested for the selected strategy?

Minor:

- Intro:
  - Rather than describing your methods, results, and conclusions in the final paragraphs of the introduction, would recommend stating your hypotheses and leaving these other pieces (except for some general methods) for the appropriate sections.
- Results
  - First two paragraphs of the results seem like introduction and methods.
- Discussion

- Another limitation is relying on EHR based ICD-10 diagnostic codes. This should be included in the limitations paragraph.

Additional comments:

The authors do a phenomenal job of leveraging advanced methods and cutting-edge technology, including artificial intelligence and transformers, applied to raw polysomnographic data in a large real-world cohort of 10,000 patients to identify unique clusters using physiologic parameters and predict long-term health outcomes. While the approach is novel, there are several concerns that limit the clinical and scientific impact of the work.

- First, the transformer model is trained on three tasks that are primarily respiratory signal–based, ignoring the complex interplay between other physiologic channels, such as cardiac and limb movement data. As a result, the derived clusters appear to be predominantly driven by the apnea-hypopnea index (AHI), offering limited novelty beyond known severity-based classifications and potentially steering the field in the direction of prior epidemiologic evidence which has not been supported in randomized trials.
- All long-term outcomes are grouped together in the analysis, with endpoints trending uniformly in one direction across clusters. There is no attempt to differentiate clusters based on specific outcome patterns—e.g., some being more associated with neurological outcomes and others with cardiac events—missing an opportunity for a more nuanced understanding.
- The validation strategy is also limited. The external validation cohort only confirmed the two-cluster solution, not the five-cluster model that is emphasized in the main findings, which reduces the rigor and generalizability of the five-cluster interpretation.
- Furthermore, within the validation cohort, only heart failure and all-cause mortality were validated as outcomes, omitting other clinically relevant endpoints. This weakens the argument that the clustering is broadly predictive or clinically actionable.
- The clustering approach also fails to incorporate important clinical and behavioral variables such as medications, sleep duration, sleep timing, and lifestyle factors, all of which play a major role in sleep health and are routinely overlooked in practice. Their exclusion further limits the translational value of the clusters.
- Ultimately, while the technical implementation is strong, the resulting model identifies patients with high AHI without substantially advancing our understanding of physiologic subtypes or impacting patient care. The clustering

does not integrate symptom burden or treatment responsiveness, which are central to current precision sleep medicine.

- Despite the use of advanced AI tools, the clinical and translational contribution is modest.
