# Supplementary material for: A foundation model for sleep-based risk stratification and clinical outcomes
Source: Nat Commun. 2026 Aug 3;17:7603. doi: 10.1038/s41467-026-75326-9 (PMC13434717; doi:10.1038/s41467-026-75326-9)
Supplement: Supplementary file 2 [file 41467_2026_75326_MOESM1_ESM.pdf]

Supplementary information

Supplementary results

Baseline analysis of novel risk groups

Supplementary Data 4 presents the baseline demographic, clinical, and polysomnographic characteristics of patients across five risk groups (RG1–RG5), with sleep variable definitions provided in Supplementary Table 1. The data are organized under several subheadings: Sociodemographics, Cardiovascular Risk Factors, Cardiovascular Disease, Neurological Disorders, Other Medical History, PSG Results, Alternative Metrics, and Other Measures.

Risk Group 1 (RG1) had the largest sample size (n=3,357) with a mean age of 50.8 years. This group had a relatively balanced gender distribution, with 52.6% males. The prevalence of cardiovascular risk factors such as hypertension (59.8%), hyperlipidemia (53.5%), and type 2 diabetes (32.7%) was moderate compared to other groups. PSG results indicated mild SDB, with an average AHI of 12.4 events/hour and an arousal index of 23.8 events/hour. This group also showed moderate levels relative to other RGs of SSF and hypoxic burden.

Risk Group 2 (RG2) consisted of younger patients with a mean age of 44.0 years and the lowest percentage of males (37.6%). This group exhibited the lowest prevalence of cardiovascular risk factors: hypertension (47.5%), hyperlipidemia (37.8%), and type 2 diabetes (24.7%). PSG findings revealed the mildest SDB of all groups, with AHI of 5.4 events/hour, and the lowest arousal index (19.3 events/hour). Neurological disorders such as migraine (19.7%) and mood disorders (50.9%) were relatively more prevalent in this group.

Risk Group 3 (RG3) had patients with a mean age of 49.7 years and an intermediate gender distribution (48.7% males). This group showed higher prevalence of mood disorders (56.6%) and chronic pain (44.8%). Cardiovascular risk factors were similar to RG1, with hypertension present in 59.7% of patients. PSG results indicated mild SDB with an AHI of 11.2 events/hour and an arousal index of 22.7 events/hour.

Risk Group 4 (RG4) included older patients with a mean age of 58.6 years and the highest percentage of males among larger groups (60.8%). This group exhibited the highest prevalence of cardiovascular risk factors: hypertension (75.6%), hyperlipidemia (65.2%), and type 2 diabetes (41.8%). Cardiovascular diseases were more common, with heart failure present in 18.4% and atrial fibrillation in 15.3% of patients. PSG results showed increased SDB severity relative to lower risk groups, with an AHI of 22.7 events/hour and a significantly higher arousal index of 41.5 events/hour, suggesting more fragmented sleep.

Similar to RG4, Risk Group 5 (RG5), the smallest group (n=363), had patients with high mean age (58.6 years) and the highest proportion of males (66.4%). This group demonstrated the most severe

PSG abnormalities, with the highest AHI (37.3 events/hour) and arousal index (60.8 events/hour), consistent with severe SDB and elevated SSF. Cardiovascular diseases were most prevalent in this group, with heart failure in 21.8% and atrial fibrillation in 17.9% of patients. Despite severe PSG findings, the prevalence of some neurological disorders, such as mood disorders (51.0%), was similar to other groups.

To further evaluate the associations between risk groups and various diseases, Supplementary Table 2 and Supplementary Data 5 provide the odds ratios of comorbidities for patients in risk groups RG2 to RG5 relative to RG1, derived from logistic regression analyses adjusted for age, BMI, and relevant comorbidities (as outlined in Supplementary Data 3). These findings largely corroborate the observations from the baseline table, with the exception of RG3, which was significantly associated with increased odds for all the diseases examined. This likely reflects adjustment for the relatively younger age of individuals in this group.

In summary, the risk groups exhibited a gradient of increasing age, male predominance, cardiovascular risk factors, and severity of SDB from RG2 to RG5. RG2 was characterized by younger age, a lower prevalence of cardiovascular risks, and milder PSG findings. RG4 and RG5 included older patients with higher cardiovascular morbidity and more severe PSG abnormalities, reflecting a higher risk profile. RG1 and RG3 shared similar PSG characteristics; however, RG3 distinguished itself by being associated with a significantly higher number of comorbidities after adjusting for demographic factors.

External validation of the two-cluster solution

We further evaluated a simplified two-cluster solution derived from our risk stratification pipeline, which identified a proxy classifier based on standard PSG metrics (e.g., AHI, oxygen desaturation, arousal indices, etc.; detailed in Supplementary Table 4). This proxy enables risk group assignment ($RG1^+$ and $RG2^+$) without requiring the full deep learning pipeline, improving clinical applicability.

Supplementary Figure 6 displays Kaplan-Meier survival curves for the two-cluster solution for all-cause mortality (panels A and B) and incident heart failure (panels C and D), with panels A and C from our propensity score-adjusted STARTLIT-10k clinical cohort and panels B and D from the independent SHHS cohort. Analyses were adjusted for age and gender, consistent with prior SHHS methodologies.

In the clinical cohort, the two-cluster solution demonstrated strong prognostic value. For all-cause mortality (panel A), $RG2^+$ showed significantly worse survival (log-rank $p = 1.1\times10^{-42}$ vs. $RG1^+$), with an age/gender-adjusted Cox hazard ratio (HR) of 1.90 (95% CI = 1.55–2.34, $p = 1.1\times10^{-9}$). Similarly, for heart failure (panel C), $RG2^+$ was associated with reduced event-free survival (log-rank $p = 2.7\times10^{-14}$ vs. $RG1^+$; adjusted HR = 1.45, 95% CI = 1.16–1.83, $p = 0.0015$).

External validation in SHHS confirmed these associations, despite variations in PSG channels and sampling rates. For all-cause mortality (panel B), $RG2^+$ exhibited significantly poorer outcomes (log-rank $p = 2.6\times10^{-12}$ vs. $RG1^+$; adjusted HR = 1.72, 95% CI = 1.34–2.22, $p = 2.5\times10^{-5}$). For incident heart failure (panel D), unadjusted analysis indicated a difference (log-rank $p = 0.004$ vs. RG1), though the adjusted HR did not reach statistical significance (HR = 1.43, 95% CI = 0.88–2.33, $p = 0.148$).

These results align with the multi-cluster findings, reinforcing the pipeline's robustness across cohorts. In contrast to traditional AHI-based analyses in SHHS, which linked outcomes primarily to severe sleep-disordered breathing (AHI ≥30) and often with sex-specific effects (as reported in the original SHHS analyses, e.g., heart failure associations limited to men), our two-cluster proxy identified significantly increased risks without such limitations, supporting its utility for broader population screening.

Supplementary methods

Data quality assessment

Sample records involving study type that was not manually confirmed as PSG were excluded. This exclusion encompassed split studies, home sleep apnea tests, multiple sleep latency tests, maintenance of wakefulness tests, and other non-PSG studies in which sleep was intentionally or artificially altered.

Data quality assessment for the 10,000 files was performed using data from six referenced channels. In an ideal setup, odd-numbered channels, positioned on the left side of the scalp, were referenced by 'M1', while even-numbered channels, positioned on the right side, were referenced by 'M2'. In rare cases of reference issues, the reference from the opposite side was employed. The electroencephalography (EEG) channels utilized were 'F3', 'F4', 'C3', 'C4', 'O1', and 'O2'[30].

A high-pass filter with a passband frequency of 1 Hz and a notch filter with a stop-band of 57.5–62.5 Hz was applied to all recordings using a zero-phase forward and reverse digital filter. Waveforms in each channel were divided into 1-second epochs, and each epoch was checked for artifacts using a previously validated machine learning method by Levitt *et al.* [31]. Three separate artifact detection scans were applied to each file, corresponding to the three non-rapid eye movement sleep stages: N1, N2, and N3. Rapid eye movement (REM) sleep was excluded due to the presence of ocular artifacts. Each scan started at the first occurrence of the respective sleep stage in the file and continued for a duration of two minutes.

In each sleep stage, the percentage of clean files was determined based on the artifact detection algorithm's results. A file was labeled as clean if it had at least four clean channels out of the six EEG channels, with each channel considered clean if it had at least 60% artifact-free data. The percentages of clean files for N1, N2, and N3 sleep stages were 90%, 88%, and 91%, respectively. A total of 267

files that were not artifact-free in all three sleep stages were excluded. No major demographic differences were observed between the excluded samples and the ones that were kept.

Polysomnogram features

The aggregated PSG metrics were extracted from the structured HTML format of the final sleep study report submitted by the provider to the EMR. The metrics parsed from the original file include total and REM AHI, arousal index, obstructive apneas, central apneas, hypopneas, mean oxygen saturation, minimum and maximum SpO2, sleep time with oxygen saturation less than 90% SpO_2, maximum $EtCO_2$, percentage of sleep time in each sleep stage, total sleep time, total REM time, and snoring. All conventional PSG variables were computed after scoring by Nihon Kohden's Polysmith 12 software. To enrich the analyses, the following alternative metrics were computed: sleep apnea-specific hypoxic burden,[32] sleep apnea-specific pulse-rate response (Delta HR),[33] lung to finger circulation time,[34] and SSF, which is defined herein and calculated as the normalized power in the "fast" frequency range of the hypnogram's power spectral density (PSD), specifically transitions occurring faster than 10 minutes.

Providers are instructed to remove text variables reporting zero values in the final PSG report if the value was not measurable. This may result in missing values that should be zero. With regard to data cleanliness and pre-processing, missing values were imputed where appropriate to address clinical data incompleteness. The following corrections were applied: sleep time with oxygen saturation $<O_2$ under 90% was set to 0 if the minimum oxygen saturation was above 90%. If the central apnea index (CAI) was zero, the number of central apneas was set to zero. To ensure accuracy, the reported AHI total was validated by recomputing the sum of individual counts of respiratory events (obstructive apneas, central apneas, mixed apneas, and hypopneas) based on total sleep time. In case of missing respiratory events and AHI total was zero, individual respiratory events counts were set to zero. When total sleep time was zero, the AHI total was set to N/A, as no sleep was observed.

Disease vocabulary and comorbidities

To define the disease vocabulary used in our analyses, we curated a comprehensive list of clinically significant conditions with their respective diagnosis codes (ICD-10) that are relevant to SDB, sleep architecture, and associated health risks (Supplementary Data 6). This vocabulary included disease outcomes: type 2 diabetes, hypertension, hyperlipidemia, heart failure (HF), atrial fibrillation (AF), migraine, mood disorders, cognitive impairment, chronic obstructive pulmonary disease (COPD), chronic pain, GERD, chronic insomnia, and MACE which included HF, myocardial infarction, coronary artery disease (CAD), coronary artery bypass grafting, and stroke. Mood disorders included depression, anxiety, bipolar disorder, post-traumatic stress disorder, and related conditions. Cognitive impairment included cognitive deficits, amnesia, and dementia-related conditions such as Alzheimer's, Parkinson's, Lewy body disease, and vascular dementia. Chronic pain included chronic pain syndrome and fibromyalgia. These conditions were identified from diagnoses recorded in the

Electronic Health Record (Epic®) that appeared at least once in our cohort. They were selected based on both established and hypothesized relationships with sleep physiology and their relevance to long-term health outcomes, with the goal of providing a broad view of how sleep-related risk factors may influence diverse health domains.

For each disease outcome, we individually selected covariates to adjust for relevant confounders that could potentially affect the association between the risk groups and outcomes (Supplementary Data 3). All models included obesity as a covariate, highlighting its importance in cardiovascular and neurologic diseases[35]. Covariate selection was based upon biological plausibility of factors considered to be confounding influences in the respective models. Type 2 diabetes was modeled with hypertension as a covariate, reflecting the well-known role this factor plays in the development and progression of diabetes[36]. Hypertension was modeled with diabetes, AF,[37] HF, CAD,[38] and hyperlipidemia, [39] acknowledging the multifactorial nature of hypertension and its associations with both metabolic and cardiovascular conditions. Hyperlipidemia was modeled with hypertension,[39] diabetes,[40] AF,[41] HF, [42] and CAD,[43] recognizing the intertwined relationships between lipid metabolism and these cardiovascular risk factors. HF was modeled with hypertension,[44] diabetes,[45] AF,[46] CAD,[47] and hyperlipidemia,[42] as these factors collectively contribute to the development and worsening of HF. AF was modeled with hypertension,[37] diabetes,[48] HF,[46] CAD,[49] and hyperlipidemia,[41] given the strong associations between these conditions and the risk of arrhythmias. Migraine was modeled with mood disorders, reflecting the interplay between these factors in the pathophysiology of migraine and sleep disorders.[50] Mood disorders were modeled with chronic pain, recognizing how these conditions are often co-occurring and can exacerbate each other's impact on mental and physical health.[51] Cognitive impairment was modeled with mood disorders, as this factor may be implicated in the progression of cognitive decline.[52,53] COPD was modeled with HF, as obesity and HF can be major contributors to the development and severity of COPD.[54] Chronic pain was modeled with mood disorders, emphasizing how chronic pain often coexists with these conditions, influencing the overall health burden.[51] GERD was modeled with hypertension and diabetes, reflecting the well-documented associations between these conditions and the development of reflux. Chronic insomnia was modeled with chronic pain[55] and mood disorders,[56] highlighting complex interactions that contribute to the persistence of insomnia.[55,56] MACE was modeled without obesity and with hypertension, diabetes, AF, and hyperlipidemia, to best capture the risk factors pertinent to these serious outcomes. This tailored approach ensured that each model accounts for disease-specific confounders, providing a robust and nuanced framework for assessing the relationship between the identified risk groups and a wide range of clinically meaningful health outcomes.

Supplementary figures

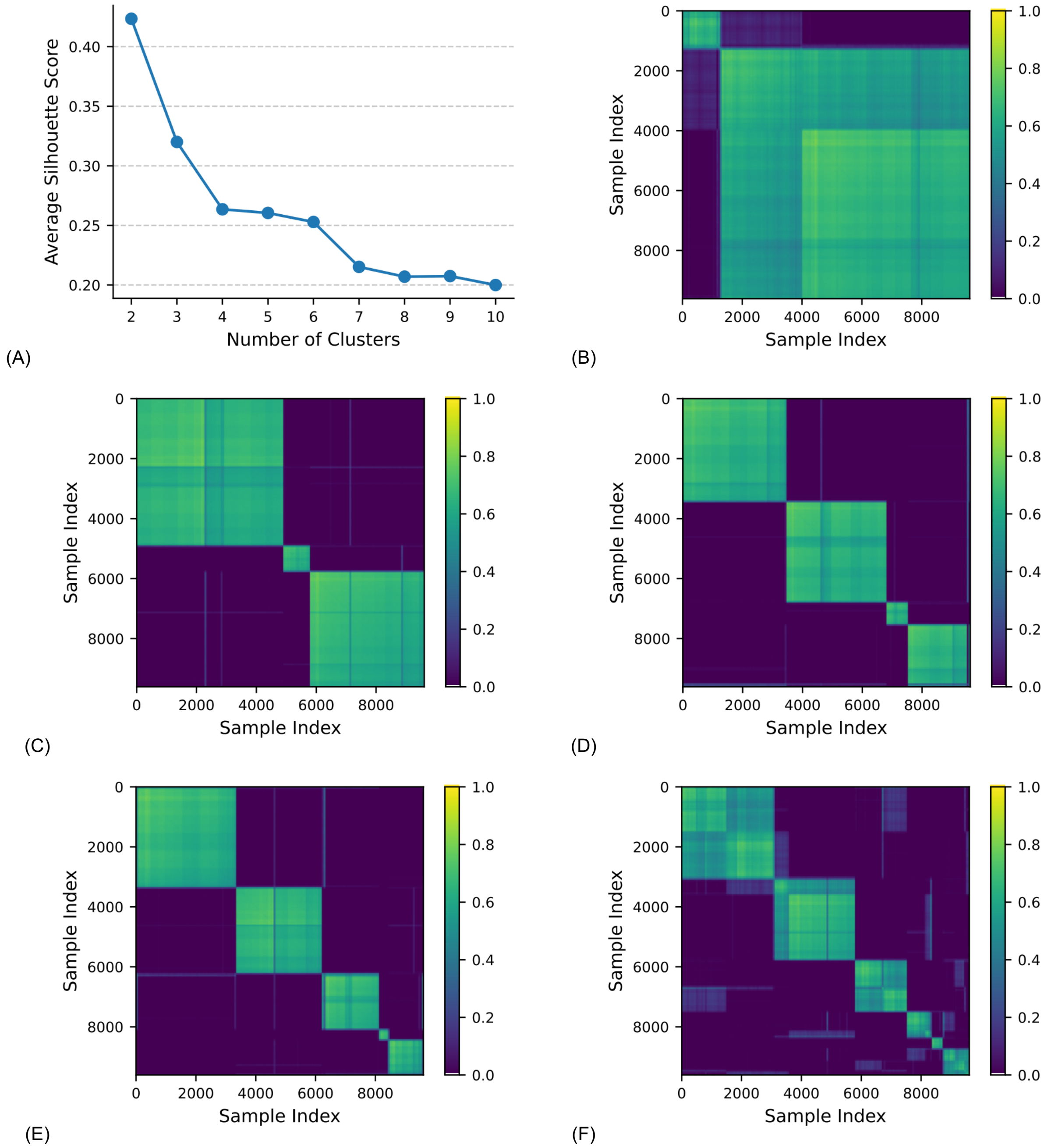

Supplementary Figure 1: Sensitivity analysis of clustering solutions across $k = 2–10$ derived using energy distance with projections on all samples. **A** Silhouette scores across $k = 2–10$. **B–F** Consensus matrices for $k = 2–6$. Panels A and E are reproduced from Figure 5 to enable comparison across all k-values.

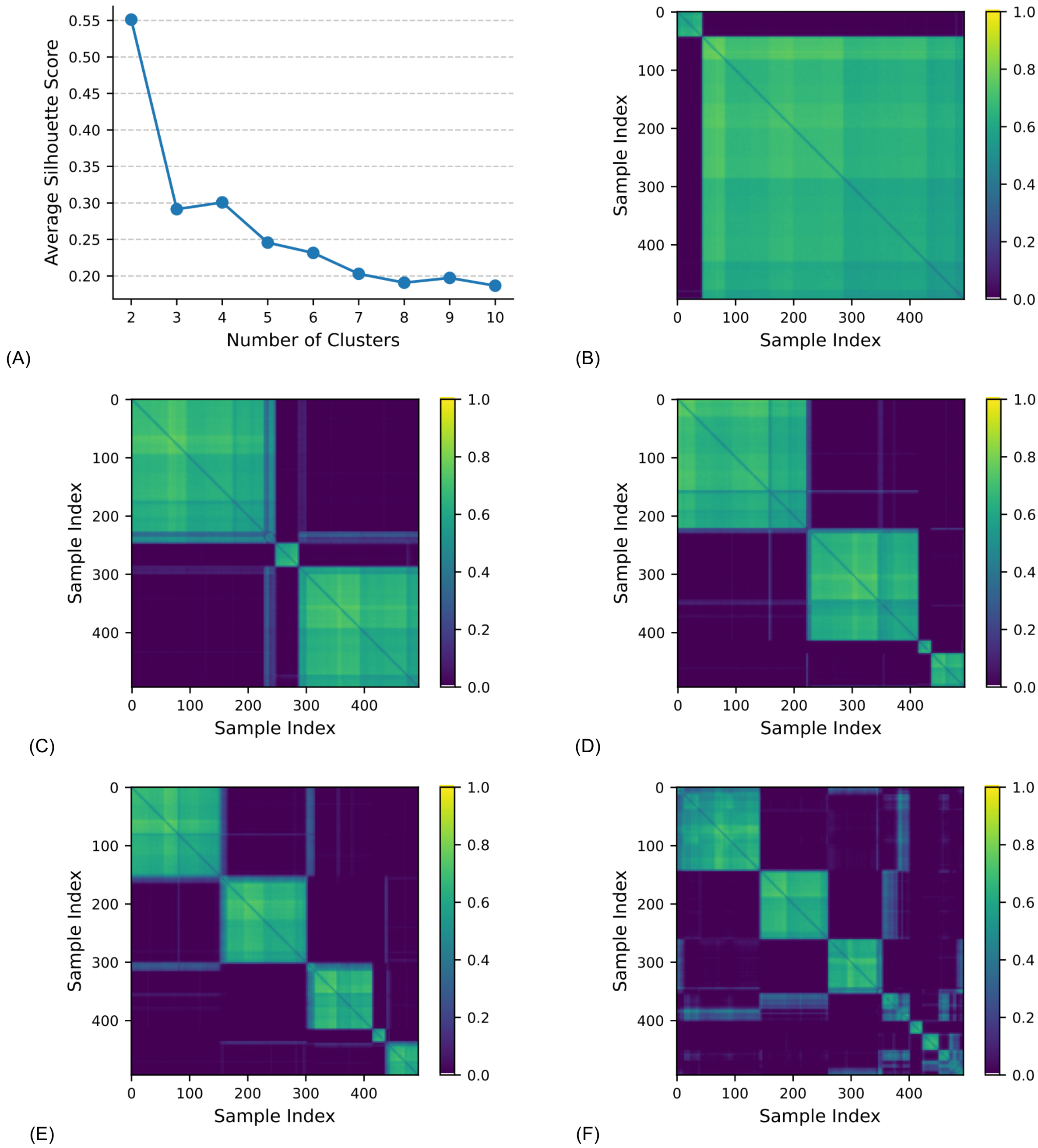

Supplementary Figure 2: Sensitivity analysis of clustering solutions across $k = 2–10$ derived using energy distance with projections on test samples. **A** Silhouette scores across $k = 2–10$. **B–F** Consensus matrices for $k = 2–6$.

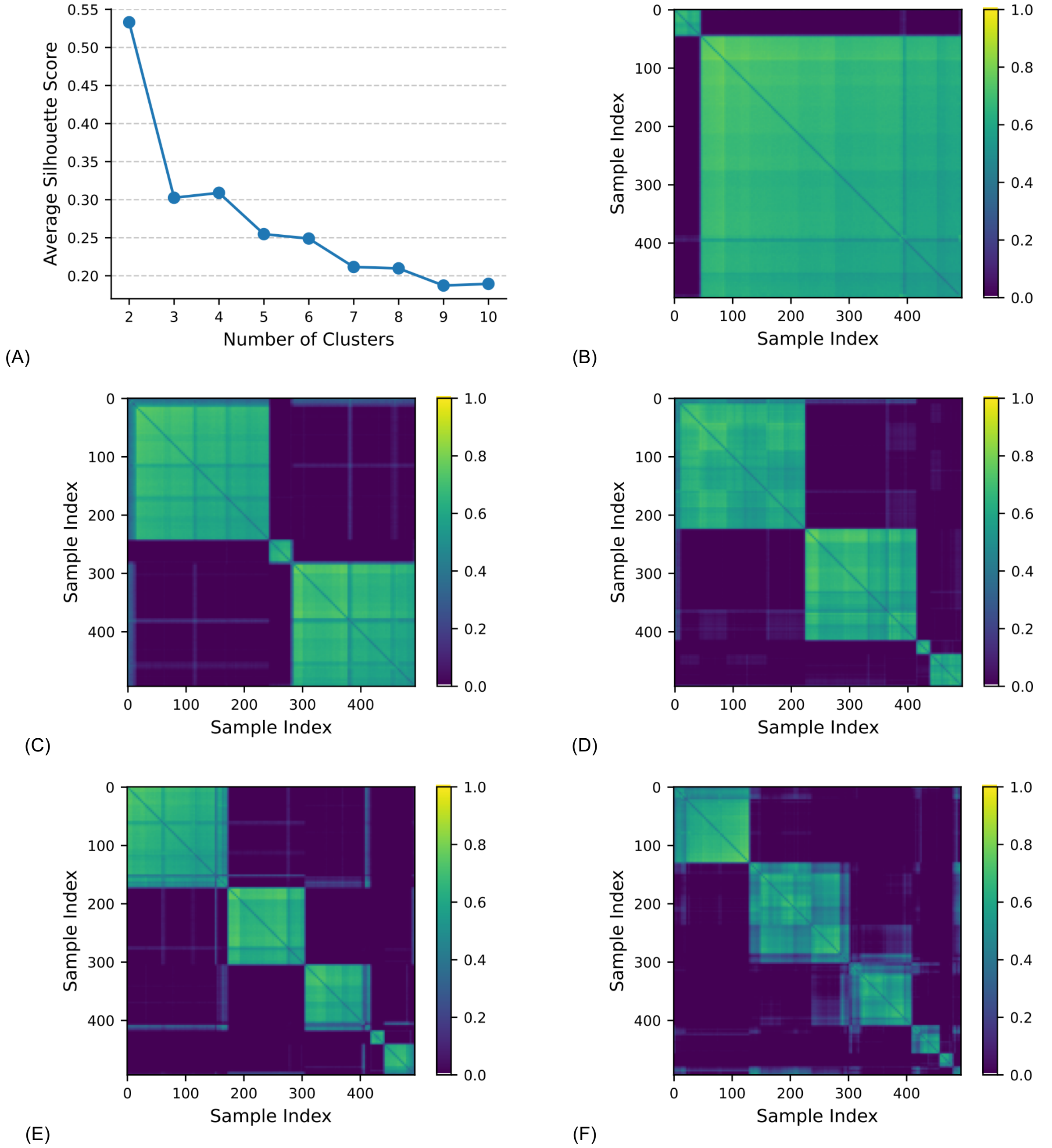

Supplementary Figure 3: Sensitivity analysis of clustering solutions across $k = 2–10$ derived using Earth mover's distance with projections on test samples. **A** Silhouette scores across $k = 2–10$. **B–F** Consensus matrices for $k = 2–6$.

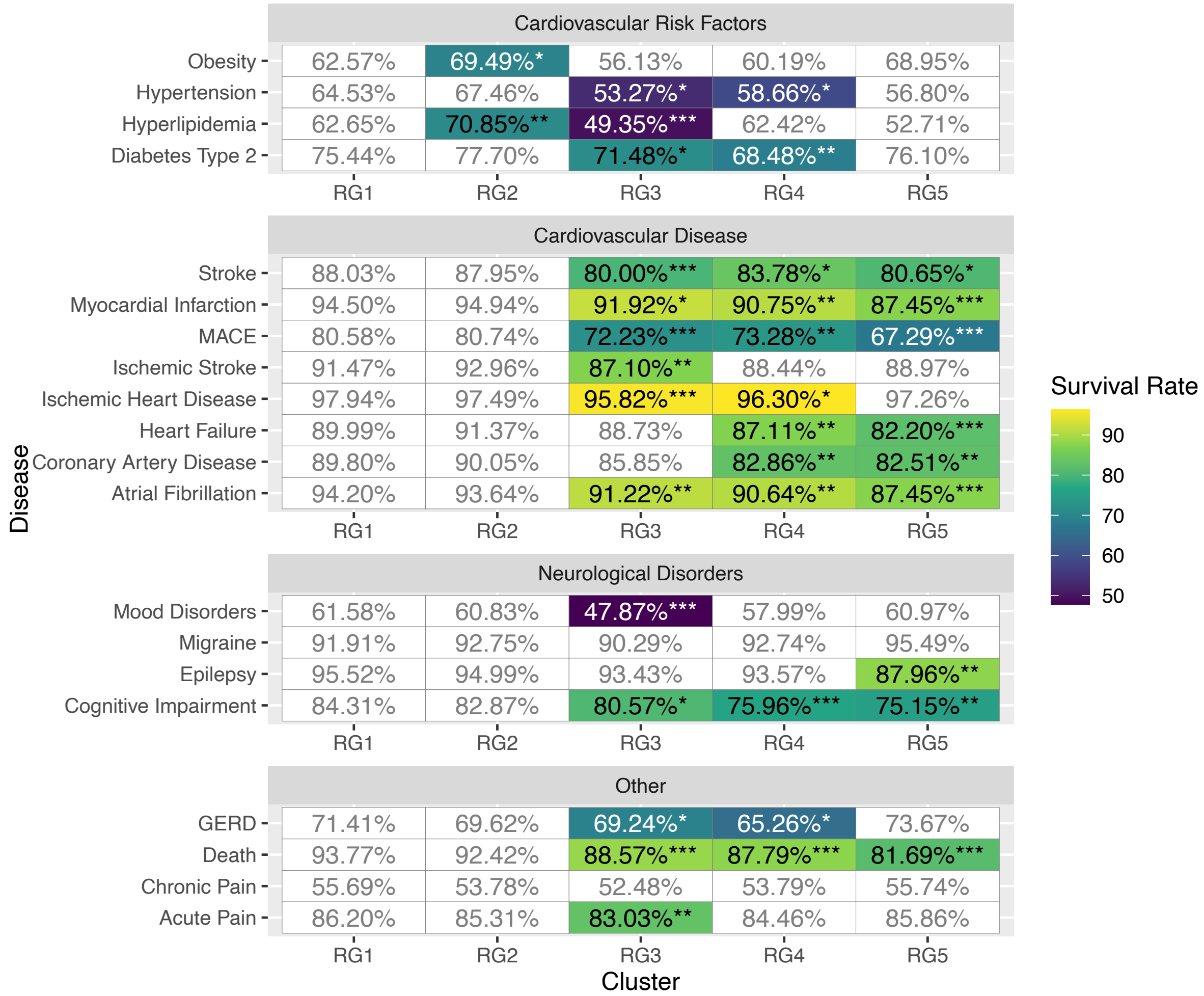

Supplementary Figure 4: Summary of 6-year disease-free clinical outcome among propensity score matched patients from different risk groups. Clinical outcomes were calculated after six years from the date of baseline PSG. Cells are color-coded for statistically significant survival rates with weighted propensity score matching such that darker color indicates worse outcome (lower survival due to disease indicated on y-axis). Significance levels are indicated with asterisks: $*p < 0.05$; $**p < 0.01$; and $***p < 0.001$. Abbreviations: MACE: Major Adverse Cardiovascular Events; GERD: Gastroesophageal Reflux Disease.

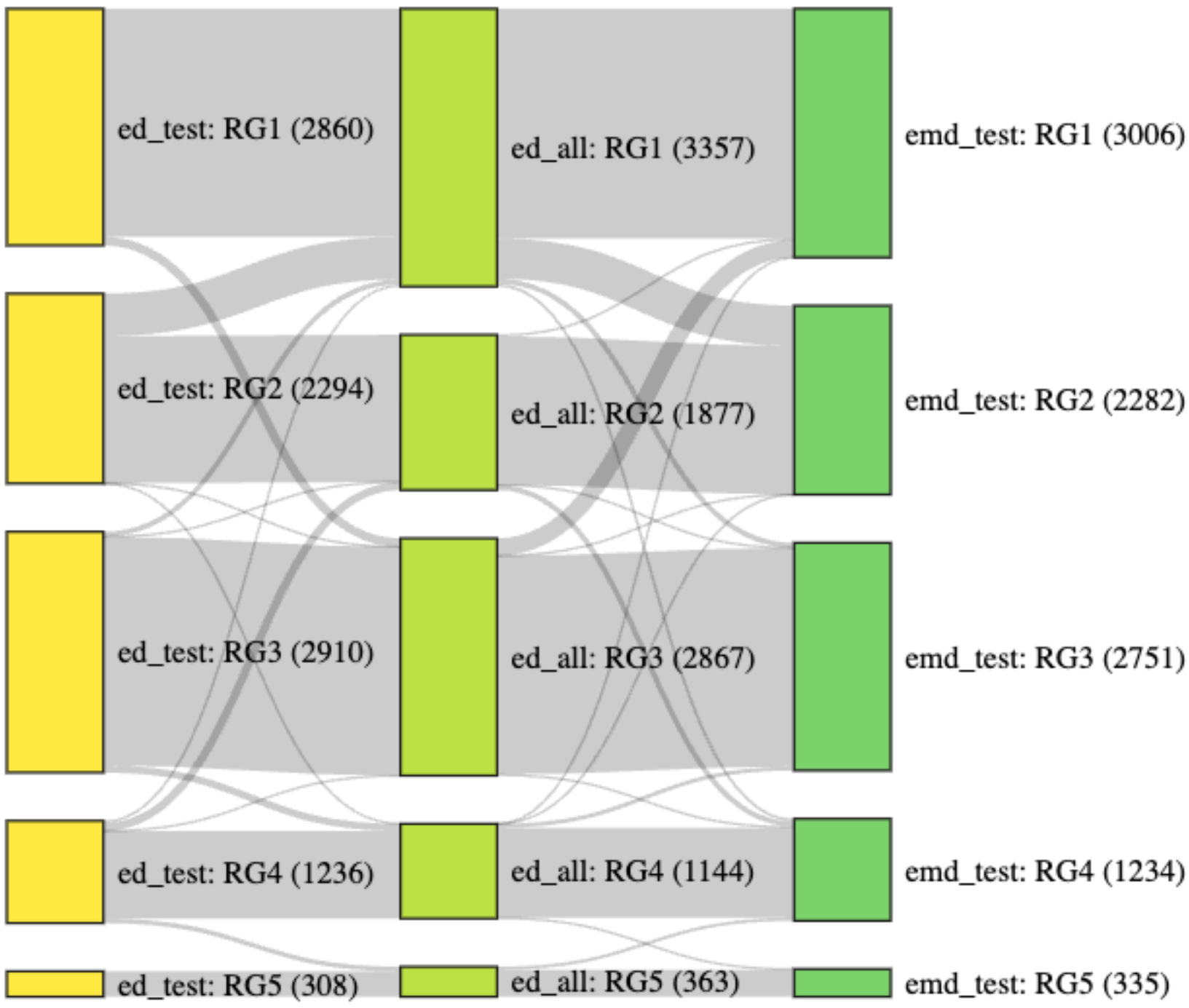

Supplementary Figure 5: Sankey diagram for the sample assignment between different five cluster solutions. The three columns correspond to five-cluster solutions derived using different distance metrics and projection strategies (Supplementary Figures 1–3).

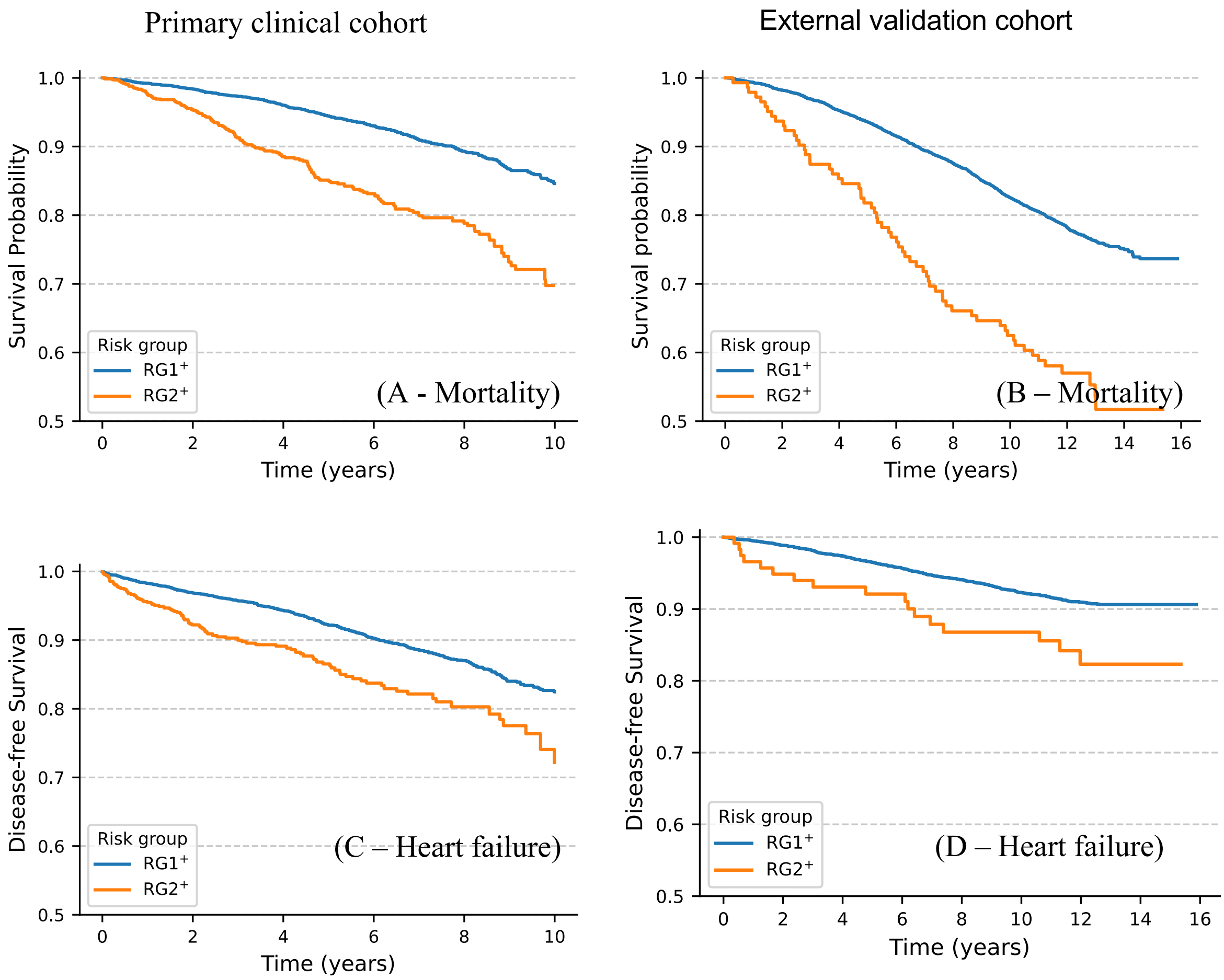

Supplementary Figure 6: Two-cluster solution validates mortality and HF associations across cohorts using standard PSG metrics. Kaplan–Meier survival curves for all-cause mortality (A, B) and incident heart failure (HF; C, D). **A, C**: STARLIT-10k clinical cohort (propensity-adjusted); **B, D**: SHHS external validation cohort. For mortality, $RG2^+$ was significantly associated with increased risk in both cohorts (STARLIT HR = 1.90, 95% CI = 1.55–2.34, p = 1.1×10⁻⁹; SHHS HR = 1.72, 95% CI = 1.34–2.22, p = 2.5×10⁻⁵) after adjusting for age and gender. For HF, $RG2^+$ showed significant separation in the clinical cohort (HR = 1.45, 95% CI = 1.16–1.83, p = 0.0015) and a similar trend in SHHS (HR = 1.43, 95% CI = 0.88–2.33, p = 0.148). These results confirm the prognostic value of the two-cluster solution and highlight its practicality, as classification can be performed directly from standard PSG metrics (see Supplementary Table 4).

Supplementary tables

Supplementary Table 1: Definitions of sleep variables.

Variable	Definition
**REM**	Rapid eye movement sleep stage
**NREM**	Non-rapid eye movement sleep stages
**N1**	Non-rapid eye movement sleep stage 1
**N2**	Non-rapid eye movement sleep stage 2
**N3**	Non-rapid eye movement sleep stage 3
**AHI, total**	Apnea hypopnea index; Respiratory events per hour of sleep

**AHI, supine**	Respiratory events per hour of supine sleep
**AHI, off-supine**	Respiratory events per hour of off-supine sleep
**REM AHI**	Respiratory events per hour of REM sleep
**NREM AHI**	Respiratory events per hour of NREM sleep
**Obstructive apnea**	≥10 seconds of ≥90% airflow reduction from pre-event baseline with continued respiratory effort
**Central apnea**	≥10 seconds of ≥90% airflow reduction from pre-event baseline without respiratory effort
**Mixed apnea**	≥10 seconds of ≥90% airflow reduction from pre-event baseline without respiratory effort for a portion of time and with respiratory effort for a portion of time
**Hypopnea**	≥10 seconds of ≥30% reduction in the nasal transducer amplitude from pre-event baseline associated with either a 4% oxygen desaturation or a 3% oxygen desaturation or arousal*
**Central apnea index**	The number of central apneas per hour of sleep
**Arousal index**	Number of arousals per hour of sleep
**Epworth Sleepiness Scale**	Self-reported questionnaire that measures daytime sleepiness based on the likelihood of dozing off in various daily situations
**Total sleep time**	Total sleep time; expressed in minutes
**Spectral Sleep fragmentation**	The normalized power in the "fast" frequency range of the hypnogram's power spectral density (PSD), specifically transitions occurring faster than 10 minutes
**Sleep apnea-specific hypoxic burden**	The area under the curve of desaturations temporally related to respiratory events
**Delta Heart Rate**	Heart rate response; change in heart rate following respiratory events
**Lung 2 finger time**	Time between the end of a respiratory event and the lowest point of oxygen desaturation

*Depending on insurer, as this is a clinical cohort.

Supplementary Table 2: Logistic Regression (Odds Ratios, 95% Confidence Intervals) of Comorbidities among Patients from Different Risk Groups.

	**Risk group**			
**Disease**	RG**2 (n=1,877)**	RG**3 (n=2,867)**	RG**4 (n=1,144)**	RG**5 (n=363)**
**Cardiovascular Risk Factors**				
**Obesity**	0.81 (0.67-0.98)(*)	2.49 (2.13-2.91)(***)	1.68 (1.36-2.06)(***)	1.59 (1.13-2.23)(**)
**Hypertension**	1.02 (0.86-1.19)	1.26 (1.10-1.45)(***)	1.22 (1.00-1.50)	1.36 (0.95-1.95)
**Hyperlipidemia**	0.77 (0.65-0.90)(**)	1.10 (0.96-1.25)	1.00 (0.83-1.21)	0.72 (0.53-0.98)(*)
**Diabetes Type 2**	0.82 (0.70-0.96)(*)	1.36 (1.20-1.53)(***)	1.19 (1.01-1.40)(*)	1.33 (1.03-1.73)(*)
**Cardiovascular Disease**				
**Stroke**	1.03 (0.80-1.32)	1.76 (1.47-2.12)(***)	1.44 (1.16-1.81)(**)	1.52 (1.08-2.13)(*)
**Myocardial Infarction**	1.02 (0.71-1.48)	2.22 (1.71-2.89)(***)	1.46 (1.07-1.99)(*)	1.85 (1.19-2.87)(**)
**MACE**	1.04 (0.86-1.26)	1.53 (1.33-1.77)(***)	1.64 (1.36-1.96)(***)	1.41 (1.05-1.89)(*)
**Ischemic Stroke**	1.06 (0.79-1.41)	1.74 (1.41-2.16)(***)	1.45 (1.13-1.87)(**)	1.53 (1.04-2.23)(*)
**Ischemic Heart Disease**	0.60 (0.28-1.25)	2.21 (1.44-3.38)(***)	1.64 (1.01-2.68)(*)	2.00 (1.04-3.85)(*)

**Heart Failure**	1.10 (0.83-1.46)	1.81 (1.47-2.23)(***)	1.86 (1.47-2.36)(***)	2.11 (1.49-2.98)(***)
**Coronary Artery Disease**	0.99 (0.79-1.25)	1.38 (1.16-1.64)(***)	1.38 (1.12-1.70)(**)	1.16 (0.84-1.60)
**Atrial Fibrillation**	0.89 (0.66-1.20)	1.47 (1.19-1.82)(***)	1.54 (1.21-1.96)(***)	1.68 (1.18-2.41)(**)
**Neurological Disorders**				
**Mood Disorders**	1.00 (0.87-1.16)	1.59 (1.40-1.80)(***)	1.58 (1.34-1.87)(***)	1.67 (1.29-2.18)(***)
**Migraine**	1.03 (0.86-1.24)	1.34 (1.15-1.56)(***)	0.96 (0.76-1.22)	0.96 (0.64-1.42)
**Epilepsy**	1.09 (0.83-1.44)	1.58 (1.27-1.98)(***)	1.62 (1.21-2.18)(**)	1.61 (1.00-2.58)(*)
**Cognitive Impairment**	1.15 (0.94-1.39)	1.74 (1.50-2.03)(***)	1.50 (1.24-1.83)(***)	1.45 (1.06-1.97)(*)
**Other**				
**GERD**	0.98 (0.85-1.14)	1.33 (1.18-1.49)(***)	1.15 (0.98-1.35)	1.02 (0.79-1.32)
**Chronic Pain**	0.86 (0.74-1.01)	2.45 (2.16-2.77)(***)	1.61 (1.37-1.90)(***)	1.45 (1.11-1.89)(**)
**Acute Pain**	0.58 (0.41-0.82)(**)	2.71 (2.18-3.38)(***)	1.57 (1.15-2.14)(**)	1.25 (0.73-2.15)

Disease abbreviations: MACE: Major Adverse Cardiovascular Events; GERD: Gastroesophageal Reflux Disease. Odds ratios were estimated using multivariable logistic regression models with risk group 1 as the reference group and adjustment for age, body mass index, gender, and outcome-specific comorbidities. Values are reported as odds ratio (95% confidence interval). Asterisks denote two-sided Wald *z*-test *p* values (**p*<0.05; ***p*<0.01; ****p*<0.001). No multiple-comparison adjustment was applied. Exact *p* values and Wald *z* statistics are provided in Supplementary Data 5.

Supplementary Table 3: Hazard ratios of incident diseases and all-cause mortality among patients from different risk groups after propensity score matching.

Disease	RG2	RG3	RG4	RG5
**Cardiovascular Risk Factors**				
**Obesity**	0.79 (0.67-0.92)**	1.10 (0.93-1.30)	0.82 (0.66-1.02)	0.90 (0.61-1.33)
**Hypertension**	1.08 (0.90-1.30)	1.16 (0.94-1.43)	1.26 (0.94-1.69)	1.31 (0.79-2.16)
**Hyperlipidemia**	0.74 (0.63-0.88)***	1.27 (1.05-1.53)*	1.02 (0.79-1.32)	1.12 (0.77-1.63)
**Diabetes Type 2**	0.93 (0.79-1.10)	1.33 (1.11-1.59)**	1.38 (1.11-1.72)**	1.05 (0.70-1.57)
**Cardiovascular Disease**				
**Stroke**	1.12 (0.92-1.35)	1.40 (1.14-1.72)**	0.97 (0.75-1.27)	1.31 (0.87-1.95)
**Myocardial Infarction**	1.17 (0.88-1.55)	1.42 (1.06-1.90)*	1.43 (1.03-1.99)*	1.92 (1.19-3.12)**
**MACE**	1.12 (0.95-1.33)	1.35 (1.13-1.62)***	1.23 (0.96-1.57)	1.56 (1.09-2.24)*
**Ischemic Stroke**	0.95 (0.75-1.21)	1.32 (1.03-1.68)*	1.01 (0.74-1.38)	1.02 (0.61-1.70)
**Ischemic Heart Disease**	1.05 (0.68-1.61)	2.01 (1.37-2.95)***	1.40 (0.86-2.30)	1.28 (0.56-2.97)
**Heart Failure**	1.03 (0.82-1.29)	1.07 (0.83-1.36)	1.23 (0.93-1.62)	1.53 (1.03-2.27)*
**Coronary Artery Disease**	1.03 (0.83-1.28)	1.15 (0.91-1.44)	1.29 (0.98-1.70)	1.44 (0.93-2.22)
**Atrial Fibrillation**	1.12 (0.85-1.47)	1.35 (1.02-1.80)*	1.43 (1.04-1.97)*	1.96 (1.24-3.10)**
**Neurological Disorders**				

**Mood Disorders**	1.03 (0.88-1.21)	1.39 (1.18-1.63)***	1.16 (0.95-1.43)	1.24 (0.89-1.73)
**Migraine**	0.81 (0.62-1.06)	1.16 (0.88-1.53)	1.06 (0.75-1.51)	0.75 (0.40-1.40)
**Epilepsy**	1.07 (0.78-1.47)	1.09 (0.77-1.56)	1.28 (0.86-1.92)	1.96 (1.16-3.32)*
**Cognitive Impairment**	1.13 (0.95-1.35)	1.17 (0.96-1.42)	1.35 (1.08-1.68)**	1.61 (1.16-2.24)**
**Other**				
**GERD**	1.05 (0.89-1.25)	1.20 (1.00-1.43)*	1.36 (1.10-1.69)**	0.92 (0.62-1.37)
**Death**	1.49 (1.19-1.87)***	1.54 (1.19-2.00)**	1.67 (1.28-2.19)***	2.49 (1.77-3.50)***
**Chronic Pain**	1.03 (0.91-1.16)	1.06 (0.92-1.22)	0.99 (0.84-1.18)	0.98 (0.74-1.29)
**Acute Pain**	1.06 (0.88-1.27)	1.36 (1.12-1.66)**	1.14 (0.89-1.46)	1.08 (0.71-1.62)

Hazard ratios were estimated using propensity-matched Cox proportional hazards models with risk group 1 as the reference group and adjustment for age, body mass index, gender, and outcome-specific comorbidities. Values are reported as hazard ratio (95% confidence interval). Asterisks denote two-sided Wald-test *p* values (* $p < 0.05$; ** $p < 0.01$; *** $p < 0.001$). No multiple-comparison adjustment was applied. Exact *p* values for the propensity-matched Cox analyses are provided in Supplementary Data 2.

Supplementary Table 4: Classification of risk groups using standard sleep measures.

Model accuracy	2-cluster solution	3-cluster solution	4-cluster solution	5-cluster solution
**XGB w/ top-1 features**	93.0%	54.6%	41.6%	41.7%
**XGB w/ top-5 features**	94.1%	67.8%	56.3%	56.2%
**Top-5 features**	SSF<br>Total sleep time<br>AHI total<br>Total REM time<br>Total NREM time	SSF<br>Total sleep time<br>Hypoxic burden<br>Total N3 time<br>AHI total	SSF<br>Total sleep time<br>AHI total<br>Hypoxic burden<br>Mean oxygen saturation	SSF<br>Total sleep time<br>AHI total<br>Hypoxic burden<br>Mean oxygen saturation

The top-5 features are in order of their importance.

Abbreviations: SSF: spectral sleep fragmentation, AHI: apnea hypopnea index, XGB: XGBoost, N3: non-rapid eye movement sleep stage 3, NREM: non-rapid eye movement sleep stage, REM: rapid eye movement sleep stage.
