# Supplementary material for: A foundation model for sleep-based risk stratification and clinical outcomes
Source: Nat Commun. 2026 Aug 3;17:7603. doi: 10.1038/s41467-026-75326-9 (PMC13434717; doi:10.1038/s41467-026-75326-9)
Supplement: Supplementary file 3 [file 41467_2026_75326_MOESM2_ESM.pdf]

Description of Additional supplementary files

Supplementary Data 1: Complete Cox proportional hazards regression results for incident disease and all-cause mortality outcomes across embedding-derived risk groups, including hazard ratios, confidence intervals, Wald statistics, and two-sided p values.

See file: SupplementaryData1.xlsx. Multi-sheet Excel workbook containing the complete Cox model results for each endpoint, with one worksheet per outcome. Each sheet reports covariates, regression coefficients, hazard ratios, standard errors, Wald z statistics, exact two-sided p values, and 95% confidence intervals.

Supplementary Data 2: Propensity score matching analysis results and matched cohort characteristics used for survival analyses across embedding-derived risk groups.

See file: SupplementaryData2.xlsx. Multi-sheet Excel workbook containing the complete weighted Cox model results for each endpoint after inverse probability of treatment weighting and trimming, with one worksheet per outcome. Each sheet reports covariates, regression coefficients, hazard ratios, standard errors, Wald z statistics, exact two-sided p values, and 95% confidence intervals.

Supplementary Data 3: Disease-specific comorbidity covariates used for adjustment in logistic regression and Cox proportional hazards models.

See file: SupplementaryData3.csv. CSV file defining the disease-specific comorbidities and clinical covariates used for multivariable adjustment in the logistic and Cox proportional hazards models, with one row per clinical outcome.

Supplementary Data 4: Demographic, clinical, polysomnographic, and derived sleep characteristics of patients stratified by embedding-derived risk groups.

See file: SupplementaryData4.xlsx. Excel workbook containing the full baseline cohort characteristics for patients assigned to risk groups RG1–RG5, organized by sociodemographics, cardiovascular risk factors, cardiovascular disease, neurologic disorders, other medical history, conventional PSG variables, alternative sleep metrics, and additional derived measures.

Supplementary Data 5: Complete baseline logistic regression analyses of prevalent disease associations across embedding-derived risk groups, including coefficients, standard errors, confidence intervals, Wald statistics, and two-sided p values.

See file: SupplementaryData5.xlsx. Multi-sheet Excel workbook containing the complete logistic regression results for each baseline disease, with one worksheet per outcome. Each sheet reports covariates, regression coefficients, odds ratios, standard errors, Wald z statistics, exact two-sided p values, and 95% confidence intervals.

Supplementary Data 6: Curated disease vocabulary, diagnosis definitions, and ICD-based mappings used to identify clinical outcomes and comorbidities from electronic medical records.

See file: SupplementaryData6.xlsx. Excel workbook containing the structured disease vocabulary, grouped clinical concepts, lexical variants, and composite endpoint definitions used for electronic medical record parsing and clinical outcome adjudication.
