# Supplementary material for: A foundation model for sleep-based risk stratification and clinical outcomes
Source: Nat Commun. 2026 Aug 3;17:7603. doi: 10.1038/s41467-026-75326-9 (PMC13434717; doi:10.1038/s41467-026-75326-9)
Supplement: Supplementary file 4 [file 41467_2026_75326_MOESM9_ESM.pdf]

nature portfolio

Corresponding author(s): DAPR: NCOMMS-25-23830A-Z

Last updated by author(s): Apr 24, 2026

Reporting Summary

Nature Portfolio wishes to improve the reproducibility of the work that we publish. This form provides structure for consistency and transparency in reporting. For further information on Nature Portfolio policies, see our Editorial Policies and the Editorial Policy Checklist.

Statistics

For all statistical analyses, confirm that the following items are present in the figure legend, table legend, main text, or Methods section.

n/a	Confirmed	
☐	☒	The exact sample size (*n*) for each experimental group/condition, given as a discrete number and unit of measurement
☐	☒	A statement on whether measurements were taken from distinct samples or whether the same sample was measured repeatedly
☐	☒	The statistical test(s) used AND whether they are one- or two-sided<br>*Only common tests should be described solely by name; describe more complex techniques in the Methods section.*
☐	☒	A description of all covariates tested
☐	☒	A description of any assumptions or corrections, such as tests of normality and adjustment for multiple comparisons
☐	☒	A full description of the statistical parameters including central tendency (e.g. means) or other basic estimates (e.g. regression coefficient) AND variation (e.g. standard deviation) or associated estimates of uncertainty (e.g. confidence intervals)
☐	☒	For null hypothesis testing, the test statistic (e.g. *F*, *t*, *r*) with confidence intervals, effect sizes, degrees of freedom and *P* value noted<br>*Give P values as exact values whenever suitable.*
☒	☐	For Bayesian analysis, information on the choice of priors and Markov chain Monte Carlo settings
☐	☒	For hierarchical and complex designs, identification of the appropriate level for tests and full reporting of outcomes
☐	☒	Estimates of effect sizes (e.g. Cohen's *d*, Pearson's *r*), indicating how they were calculated

Our web collection on statistics for biologists contains articles on many of the points above.

Software and code

Policy information about availability of computer code

Data collection	Raw physiological signals and corresponding epoch-by-epoch examination annotations collected using Nihon Kohden's Polysmith software (version 12).
Data analysis	Survival analyses were conducted with the open source lifelines package (v0.28.0). Statistical modeling, clustering, and related analyses used scikit learn (v1.7.2), SciPy (v1.10.1), NumPy (v1.26.4), and pandas (v2.0.3), with visualization in Matplotlib (v3.10.6). The foundation model development and training pipeline for polysomnography used PyTorch (>=2.1) and transformers (>=4.44). No commercial or proprietary analysis software is documented; analyses were executed via custom Python scripts in the study codebase. The code and sample data is documented here https://zenodo.org/records/18408622?token=eyJhbGciOiJIUzUxMiJ9.eyJpZCI6ImJmYjZmNTI4LTEyZDEtNDliYi04ZTMyLTBjNTk2MGNkODc4MCIsImRhdGEiOnt9LCJyYW5kb20iOiI5ZDYzMDVhYzViMTdhNzcxY2NkZDMzZTBkOWUyYzBmMCJ9.tB9Dfil2rUGio4DNlBh9YXEyw8gtJ6FW-nb5Y-otlUG6BTN5InHaypRzRHF7RL5qD243hegkXbZfuZ1AWyku6w .

For manuscripts utilizing custom algorithms or software that are central to the research but not yet described in published literature, software must be made available to editors and reviewers. We strongly encourage code deposition in a community repository (e.g. GitHub). See the Nature Portfolio guidelines for submitting code & software for further information.

Data

Policy information about availability of data

All manuscripts must include a data availability statement. This statement should provide the following information, where applicable:

- Accession codes, unique identifiers, or web links for publicly available datasets
- A description of any restrictions on data availability
- For clinical datasets or third party data, please ensure that the statement adheres to our policy

The data that support the findings of this study are derived from two sources. Data from the Sleep Heart Health Study (SHHS) is promptly available for reproducible purposes through SleepData.org, subject to registration and approval under the repository's data access policies. The Cleveland Clinic dataset was used under license for this study and is not publicly available due to institutional and ethical restrictions imposed by the Cleveland Clinic Institutional Review Board (IRB), which prohibits sharing data outside the approved study team. Access to these data requires submission of a new application for IRB approval at the Cleveland Clinic Foundation, along with execution of a data use agreement. Requests for access may be made by qualified researchers for purposes consistent with the original study objectives and ethical approvals. Requests should be directed to Dr. Matheus Araujo at limadim@ccf.org providing details related to the purpose. An initial response is typically provided within one week from Dr. Araujo. However, all requests must be reviewed by the Cleveland Clinic IRB and are subject to institutional approval, which typically requires 12-15 weeks. If approved, access will be granted for a limited period in accordance with the terms of the data use agreement.

Research involving human participants, their data, or biological material

Policy information about studies with human participants or human data. See also policy information about sex, gender (identity/presentation), and sexual orientation and race, ethnicity and racism.

Reporting on sex and gender	Gender was considered in the study design and analysis. Gender was obtained from the demographic "gender" field of the STARLIT-10K clinical dataset and reflects patient-reported gender identity recorded in the electronic medical record. The findings are intended to apply across genders. The study population included similar numbers of participants recorded as female and male (approximately 50% female, 50% male).
Reporting on race, ethnicity, or other socially relevant groupings	Race and ethnicity were included as socially constructed variables and reported to characterize the study population. Race and ethnicity data were self-reported by patients and obtained from electronic health records using standard clinical categorization. Race was recorded as White (44.0%), Black (34.0%), Asian (5.3%), and multiracial (14.8%). Ethnicity was recorded separately from race, with 14.6% of participants identifying as Hispanic or Latino. Race and ethnicity were not used as proxies for other socially constructed variables, such as socioeconomic status, nor were they interpreted as biological determinants.
Population characteristics	Age, gender, race, and ethnicity were considered as population characteristics. Gender was obtained from the demographic "gender" field of the STARLIT-10K clinical dataset derived from the electronic medical record. Race and ethnicity were self-reported by patients and recorded in the electronic health record using standard clinical categorization.
Recruitment	Participants were drawn from the IBM–Cleveland Clinic Sleep Study (STARLIT-10K), a retrospective clinical cohort comprising in-laboratory polysomnography (PSG) studies conducted at the Cleveland Clinic between 2012 and 2022. The cohort includes patients referred for clinically indicated PSG, with linked clinical, demographic, and outcome data obtained from electronic health records. Sampling criteria were applied to promote age diversity, temporal coverage, and representation of underrepresented groups within the cohort. As a clinic-based retrospective study, the cohort is subject to selection bias, as participants were limited to individuals referred for in-laboratory sleep evaluation. Most participants were located in the Cleveland, Ohio region, introducing potential geographic bias that may limit generalizability. Data collection overlapped with the COVID-19 pandemic, which may have influenced referral patterns, healthcare access, and disease presentation during certain periods.
Ethics oversight	Cleveland Clinic Institutional Review Board approved this study.

Note that full information on the approval of the study protocol must also be provided in the manuscript.

Field-specific reporting

Please select the one below that is the best fit for your research. If you are not sure, read the appropriate sections before making your selection.

☒ Life sciences ☐ Behavioural & social sciences ☐ Ecological, evolutionary & environmental sciences

For a reference copy of the document with all sections, see nature.com/documents/nr-reporting-summary-flat.pdf

Life sciences study design

All studies must disclose on these points even when the disclosure is negative.

Sample size	Originally, 10000 polysomnograms from 9661 patients were considered for analysis from IBM-Cleveland Clinic Sleep Study (STARLIT-10K). After data cleaning, 9608 polysomnograms from 9297 patients were used for clustering analysis.
Data exclusions	Polysomnograms that were not artifact-free in all three sleep stages were excluded.

Replication	Findings were replicated in an independent external cohort, the Sleep Heart Health Study (SHHS).
Randomization	Not applicable. Participants were not randomized, as analyses were conducted on retrospective polysomnography and longitudinal electronic health record data.
Blinding	Blinding was not relevant to this work since it was a retrospective analysis.

Reporting for specific materials, systems and methods

We require information from authors about some types of materials, experimental systems and methods used in many studies. Here, indicate whether each material, system or method listed is relevant to your study. If you are not sure if a list item applies to your research, read the appropriate section before selecting a response.

Materials & experimental systems

n/a	Involved in the study
☒	☐ Antibodies
☒	☐ Eukaryotic cell lines
☒	☐ Palaeontology and archaeology
☒	☐ Animals and other organisms
☐	☒ Clinical data
☒	☐ Dual use research of concern
☒	☐ Plants

Methods

n/a	Involved in the study
☒	☐ ChIP-seq
☒	☐ Flow cytometry
☒	☐ MRI-based neuroimaging

Clinical data

Policy information about clinical studies

All manuscripts should comply with the ICMJE guidelines for publication of clinical research and a completed CONSORT checklist must be included with all submissions.

Clinical trial registration	This study was a retrospective, observational analysis of clinical polysomnography and electronic health record data and did not involve any prospective intervention or assignment to treatment groups. Accordingly, it does not meet the definition of a clinical trial, and clinical trial registration and CONSORT reporting are not applicable.
Study protocol	This study was not a clinical trial. It was a retrospective observational study conducted under an Institutional Review Board–approved protocol at the Cleveland Clinic (IRB#23-409). No prospective study protocol or trial registration was required.
Data collection	This was a retrospective study. Data were collected between January 2012 and December 2022 from in-laboratory polysomnography studies conducted at Cleveland Clinic Sleep Disorders Center sites, with associated clinical, demographic, and outcome data obtained from the electronic medical record.
Outcomes	Study outcomes included polysomnographic metrics, clinical diagnoses, and all-cause mortality. Polysomnographic metrics were extracted from finalized sleep study reports. Clinical outcomes were identified from a predefined vocabulary of diagnoses and corresponding ICD codes in the electronic medical record, established through medical consensus. Longitudinal follow-up and all-cause mortality were ascertained by integrating electronic medical record updates with the Ohio Death Index and the Social Security Death Index.

Plants

Seed stocks	n/a
Novel plant genotypes	n/a
Authentication	n/a
